# Synergistic antitumor activity of sorafenib and the NUPR1 inhibitor LZX-2-73 in multiple cancer models

**DOI:** 10.1038/s41419-025-08178-8

**Published:** 2025-11-17

**Authors:** Xi Liu, Matías Estaras, Emma Cosialls, Ling Peng, Patricia Santofimia-Castaño, Juan Iovanna

**Affiliations:** 1 Centre de Recherche en Cancérologie de Marseille (CRCM), INSERM U1068, CNRS UMR 7258, Aix-Marseille Université and Institut Paoli-Calmettes, Parc Scientifique et Technologique de Luminy, Translational Research and Therapeutic Targets in Pancreatic Cancer, Marseille, France; 2 Aix-Marseille Université, CNRS, Centre Interdisciplinaire de Nanoscience de Marseille, UMR 7325, Parc Scientifique et Technologique de Luminy, Equipe labélisée Ligue Nationale contre le cancer, Marseille, France; 3 Hospital de Alta Complejidad El Cruce, Florencio Varela, Buenos Aires Argentina; 4 University Arturo Jauretche, Florencio Varela, Buenos Aires Argentina

**Keywords:** Translational research, Chemotherapy

## Abstract

Combination therapy in cancer treatment offers significant potential to overcome drug resistance, enhance efficacy, reduce toxicity, and expand drug indications. sorafenib, an FDA-approved multi-targeted kinase inhibitor, has demonstrated effectiveness across various cancers but currently lacks approved combination therapies. Recently, we identified LZX-2-73 as a promising drug candidate with potent anticancer activity, targeting the nuclear protein 1 (NUPR1), an emerging and promising target in cancer therapy. In this study, we report that the combination of the NUPR1 inhibitor LZX-2-73 with sorafenib produces strong synergistic anticancer effects in various cancer cell lines as well as in primary pancreatic ductal adenocarcinoma (PDAC) organoids. This combination significantly enhanced lactate dehydrogenase (LDH) release and caspase 3/7 activity, markedly induced ROS accumulation, reduced the reduced/oxidized glutathione ratio, and increased the accumulation of malondialdehyde (MDA) and lipid hydroperoxides. Collectively, the combination of LZX-2-73 and sorafenib led to a substantial increase in cell death due to massive oxidative stress. Additionally, in a pancreatic cancer xenograft mouse model, the combination of LZX-2-73 and sorafenib exhibited a synergistic anticancer effect, effectively inhibiting tumor growth. In summary, this study provides valuable insights into enhancing the anticancer activity of NUPR1 inhibitors through combination with sorafenib, offering a promising new avenue for cancer therapy and opening new indications.

## Introduction

In tumor therapy, the use of monotherapy often leads to the development of drug resistance over time. To address this challenge, combination therapy -using two or more drugs with different mechanisms of action- has proven highly effective. This approach not only helps to overcome drug resistance but also enhances therapeutic efficacy, reduces toxicity associated with high doses of a single drug, and broadens the range of conditions for which the drugs can be used [1, 2]. Common clinical combinations of antitumor drugs include the pairing of targeted anticancer agents with different mechanisms of action, combining targeted therapies with chemotherapy, integrating targeted therapies with immunotherapy, and combining immunotherapy with chemotherapy [3]. A notable example of such synergy is the combination of irinotecan and rabusertib (also known as LY260398), which has shown a powerful anticancer effect in KRAS-TP53 double-mutant colorectal cancer. Rabusertib, a selective checkpoint kinase 1/2 (CHEK1/2) inhibitor, is typically used to treat ovarian, non-small cell lung, and breast cancers [4, 5]. In the last few years, many clinical trials aimed to find synergistic effects in many types of cancers: loncastuximab tesirine with rituximab in patients with relapsed or refractory follicular lymphoma [6], inavolisib plus palbociclib-fulvestrant in PIK3CA-mutated advanced breast cancer [7], or Pembrolizumab plus lenvatinib in patients with pleural mesothelioma [8].

Sorafenib, a frontline clinical medication for HCC, inhibits tumor growth and microvasculature through antiproliferative, antiangiogenic, and/or proapoptotic effects by its multi-kinase-inhibitory activity, targeting the vascular endothelial growth factor receptors, the platelet-derived growth factor receptor, and FLT3, C-Kit, and B-RAF. However, this lack of specificity led to gastrointestinal adverse effects. In addition, sorafenib has a low bioavailability due to oral administration and fast metabolism [9]. Thus, enhancing the therapeutic effect of sorafenib, while minimizing its adverse effects, might represent a good therapeutic strategy. Original strategies, such as new nanodrug delivery systems, have implemented the activity of Sorafenib, in vivo and in vitro [10]; however, a phase II trial of sorafenib and doxorubicin in patients with advanced hepatocellular carcinoma after disease progression on sorafenib was performed, unfortunately, with no positive result [11].

Ferroptosis is an iron-dependent form of regulated cell death characterized by the accumulation of lethal lipid peroxides, distinct from apoptosis, necrosis, and other cell death modalities [12, 13]. This process is driven by oxidative stress and disrupted redox homeostasis, often involving the inactivation of the lipid repair enzyme glutathione peroxidase 4 (GPX4) or depletion of intracellular glutathione [14]. Since its formal identification in 2012, ferroptosis has garnered attention for its potential role in various pathologies, particularly in cancer, where tumor cells frequently exhibit altered iron metabolism and redox imbalance [15]. Notably, some cancer cells that are resistant to traditional forms of cell death appear susceptible to ferroptosis, making it an attractive therapeutic target [16]. Incorporating ferroptosis-inducing strategies into cancer treatment may overcome resistance mechanisms and improve outcomes in refractory tumors. Therefore, understanding the molecular underpinnings of ferroptosis is of growing importance in the context of cancer biology and therapy.

Nuclear protein 1 (NUPR1), also known as p8 and Com1, is emerging as a promising target for cancer therapy. This 82-amino acid chromatin-binding protein was first identified in 1997 in the exocrine pancreas during cellular injury caused by acute pancreatitis [17]. Subsequent research has revealed that NUPR1 is widely expressed in various cancer tissues, while it remains absent in healthy, unstressed cells. Notably, NUPR1 is overexpressed in several types of cancer cells, including pancreatic ductal adenocarcinoma (PDAC) cells [18, 19]. NUPR1 is implicated in numerous cancer-related processes, including cell cycle regulation, apoptosis [20, 21], cell migration, invasion, adhesion [22], metastasis [23], and DNA repair responses [24]. Gene inactivation of NUPR1 in different cancer cell lines, such as PDAC [22, 25], hepatocarcinoma [26], glioblastoma [27], multiple myeloma [28], osteosarcoma [29], and non-small lung cancer [30], has been shown to halt tumor growth. The role of NUPR1 in promoting the development and progression of PDAC has recently garnered significant attention, driving efforts to develop small-molecule inhibitors targeting this protein [31]. However, NUPR1 presents a unique challenge as it is an intrinsically disordered protein (IDP) without a stable three-dimensional structure [32–34]. This characteristic makes traditional structure-based high-throughput screening methods for small-molecule inhibitors unsuitable for NUPR1 [35, 36]. Despite these challenges, a combination of biochemical, biophysical, and biological techniques led to the identification of LZX-2-73, a promising NUPR1 inhibitor. LZX-2-73 demonstrates potent anticancer activity. In clinical practice, most patients with PDAC receive chemotherapy. Treating cancer cells with NUPR1 inhibitors leads to cell death through various processes, including apoptosis, necroptosis, and ferroptosis [35, 37]. This indicates that NUPR1 plays a protective role in helping cancer cells to increase resistance against stress and promoting survival against chemotherapy [38, 39]. In addition, previous studies have suggested a synergistic effect between sorafenib and NUPR1’s inhibition [26, 40], being highlighted as an important biomarker of sorafenib resistance [41, 42]. Therefore, a combination of sorafenib with LZX-2-73 might represent an interesting strategy for resistant tumors.

This study aimed to evaluate the combination of LZX-2-73 with classical chemotherapeutic agents to enhance their therapeutic efficacy. From all the drugs tested, sorafenib showed the best synergistic profile with LZX-2-73 in a wide range of cancers. The two drugs potentiate the oxidative stress, inducing massive cell death, offering a promising new approach for clinical combination therapy.

## Material and methods

### Experimental reagents and materials

Oxaliplatin, gemcitabine, paclitaxel, 5-FU, and SN38 were purchased from the Selleckchem company. Sorafenib was purchased from MedChemExpress. LZX-2-73 has been synthesized by AGVdiscovery (France). Z-VAD-FMK (Z-VAD), Necrostatin-1 (Nec-1), Ferrostatin-1 (Fer-1), N-acetyL-cysteine (NAC), were obtained from Merk.

### Cell culture and patient-derived organoids culture

Human PDAC cells MIAPaCa-2, human colorectal cancer cells HT-29, human lung carcinoma cells H1299, human breast adenocarcinoma cells MCF-7 and MDA-MB-231, human primary glioblastoma cells U87, human hepatocellular carcinoma cells HepG2 and Hep3B were cultured in Dulbecco’s modified Eagle’s medium (DMEM) from Gibco supplemented with 10% fetal bovine serum (FBS) from Biosera. Human prostate cancer cells PC-3, human lung carcinoma cells H358, human Burkitt lymphoma cells Daudi, and human acute T cell leukemia cells Jurkat were cultured in Roswell Park Memorial Institute (RPMI) 1640 medium from Gibco with 10% FBS. Cells were obtained from the American Type Culture Collection (ATCC, USA). Primary human PDAC cell lines were cultured in DMEM/F12 medium supplemented with 1.22 g/L nicotinamide, 5 g/L glucose, 5% Nu-Serum IV, 0.5% ITS+ Premix Universal Culture Supplement (containing insulin, human transferrin, and selenous acid), 1 μM dexamethasone, 10 ng/L cholera toxin, 50 nM 3,3′5-triiodo-L-thyronine, 25.2 mg/L bovine pituitary extract, and 20 μg/L epidermal growth factor. Cells were maintained at 37 °C with 5% CO_2_ in a humidified incubator.

Patient-derived organoids were obtained from a series of PDAC patients, including those with both resectable and unresectable tumors, as part of the PaCaOmics clinical trial NCT01692873. The organoids were cultured using Pancreatic Organoid Feeding Media (POFM), which consisted of Advanced DMEM/F12 with the addition of 10 mM HEPES, 1× Glutamax, and penicillin/streptomycin from Thermo Fisher. Supplements included 100 ng/ml Animal-Free Recombinant Human FGF10 and 50 ng/ml Animal-Free Recombinant Human EGF from Peprotech, 100 ng/ml Recombinant Human Noggin from Biotechne, 30% Wnt3a-conditioned medium, 10% RSPO1-conditioned medium, 10 nM human Gastrin 1, 10 mM Nicotinamide, 1.25 mM N-acetylcysteine from Sigma Aldrich, 1× B27 from Invitrogen, 500 nM A83-01, and 10.5 μM Y27632 from Tocris. The organoids were incubated at 37 °C in a 5% CO_2_ atmosphere, with the media being refreshed every 3–4 days.

### Drug combination assay

Drug combination assays were conducted to assess the synergistic effects of LZX-2-73 in combination with SN38, oxaliplatin, gemcitabine, paclitaxel, 5-FU, or sorafenib on various cancer cell lines. Cells were exposed to different doses of LZX-2-73 and the aforementioned compounds using an 11 × 7 dose matrix that included concentrations above and below their IC_50_, as determined from previous studies. Initially, cancer cells were seeded into clear 96-well plates at a density of 5000 cells per well and cultured for 24 h. Serial dilutions from the main stock solutions of each compound were used to create the dosing matrix. The range concentration for each drug was: LZX-2-73 (2–10 µM), sorafenib (0.01–50 µM), SN38 (0.001–1000 µM), oxaliplatin (0.001–1000 µM), 5-FU (0.001–1000 µM), paclitaxel (0.001–60 µM), and gemcitabine (0.062–250 µM). After seeding for 24 h, the compounds were applied to the cells in triplicate and incubated for 72 h at 37 °C with 5% CO_2_. Following incubation, PrestoBlue™ Cell Viability Reagent from Thermo Fisher Scientific was added to each well, and the plates were incubated for an additional 2 h under the same conditions. Cell viability and inhibition were quantified using the Tristar LB941 plate reader. Untreated cells served as the control. The SynergyFinder software, version 3.0, was utilized to calculate synergy scores for the drug combinations based on the HSA reference model. A synergy score below −10 indicated antagonistic interactions, scores between −10 and 10 indicated additive interactions, and scores above 10 indicated synergistic interactions [43, 44].

### Cell viability

Cell viability was determined using the PrestoBlue assay. Cells were seeded at 5000 cells per well in a 96-well plate, then treated the next day as indicated in 100 µL of medium. After 72 h of treatment, 10 µL of PrestoBlue (Thermo Fisher, A13262) was added to the medium and incubated for 2 h at 37 °C + 5% CO_2_. Then, 100 µL of medium was collected in a black plate. Cell viability and inhibition were quantified using the Tristar LB941 plate reader. Untreated cells served as the control.

### Proliferation assay

Cultures were plated at 5000 cells per well in 96-well plates and allowed to incubate for 24 h to adhere to the surface. Cells were then treated with sorafenib, LZX-2-73, or a combination of both for 72 h. During the incubation period, cell growth was monitored using the Incucyte® S3 Live-Cell Analysis System (Sartorius, Germany), capturing phase contrast images every 4 h and quantifying culture confluence with the integrated confluence algorithm. All experiments were conducted in triplicate.

### Organoid imaging

Organoids were seeded at 1000 cells/well in 96-well plates (surface: BIOFLOAT™, round base) and incubated for 2 days to form organoids. The following treatments were then applied for 72 h: PDAC056T organoids were treated with 12.5 µM sorafenib alone, 25 µM LZX-2-73 alone, or a combination of both; PDAC088T organoids were treated with 10 µM sorafenib alone, 25 µM LZX-2-73 alone, or a combination of both. Three days after drug treatment, the organoids were photographed using an EVOS cell imaging system (Invitrogen by Thermo Fisher Scientific). The diameter of the organoids was analyzed using ImageJ software.

### LDH and caspase 3/7 activity measurement

Cultures were initially plated at 5000 cells per well in 96-well plates and allowed to incubate for 24 h to adhere to the surface. Cells were then treated with sorafenib, LZX-2-73, or a combination of both for 72 h. The LDH release and caspase 3/7 activity were measured using CytoTox-ONE™ and Caspase-Glo® 3/7 (Promega, France), respectively. For LDH and caspase 3/7 activity assays, data were normalized to the cell number.

### Glutathione assay

The GSH/GSSG-Glo assay kit (V6611, Promega) was utilized according to the manufacturer’s instructions. Briefly, 5000 cells per well were plated in 96-well plates and left to grow overnight. The treatments are the same as those in the LDH release assay applied for 72 h, with each condition tested in triplicate. Measurements of total intracellular glutathione and oxidized glutathione (GSSG) were taken. To each well, the Luciferin Generation Reagent and Detection Reagent were added, mixed thoroughly, and the resulting luminescence was recorded using the Tristar multimode microplate reader. The concentrations of GSSG and total glutathione were determined using a standard curve and normalized to the cell count. The GSH/GSSG ratios were calculated with the formula: GSH/GSSG = [Total GSH - (2 × GSSG)]/GSSG.

### Lipid peroxidation determination

The MDA lipid peroxidation assay kit (ab118970, ABCAM, Cambridge, UK) was employed following the manufacturer’s instructions. To measure MDA production in MIAPaCa-2 xenografts, 10 mg of tumor tissue was utilized. For cell experiments, 5 × 10^5^ cells were seeded into 10-cm cell culture dishes and allowed to attach overnight. Treatments were performed with sorafenib, LZX-2-73, or a combination of both for 72 h and tested in triplicate. Tumor tissues and cells were collected, homogenized in a lysis solution, and centrifuged to obtain the supernatant. An equivalent amount of protein from each sample was mixed with the thiobarbituric acid solution and incubated at 95 °C for 1 h. Subsequently, the samples were cooled in an ice bath for 10 min to reach room temperature. Fluorescence measurements were acquired using a TECAN Infinite 96-plate reader with excitation/emission settings of 532/553 nm.

Cells were plated in 12-well plates at a density of 25,000 cells per well. The treatments are the same as those in the LDH release assay applied for 72 h, with each condition tested in triplicate. Subsequently, the cells were incubated in fresh medium containing 2 µM BODIPY™ 581/591 C11 (Invitrogen Molecular Probes, D3861) at 37 °C for 30 min. After incubation, the cells were washed twice with PBS. Finally, samples were imaged and analyzed with a Zeiss Axio Imager Z2 microscope.

### GPx activity assay

GPx activity was assessed using a Glutathione Peroxidase Assay Kit (ab102530, ABCAM, Cambridge, UK). The assay relies on the GPx-catalyzed oxidation of glutathione (GSH) to glutathione disulfide (GSSG), which is then converted back to GSH by glutathione reductase using NADPH. The decrease in NADPH, reflected by its oxidation to NADP^+^, serves as an indicator of GPx activity. In summary, tumor tissues were homogenized using a Dounce homogenizer, and the supernatants were collected and kept on ice after centrifugation. Following the manufacturer’s instructions, the samples were mixed with the reaction reagent, and the optical density (OD) at 340 nm was measured. Cumene hydroperoxide solution was subsequently added to the samples. The enzymatic reaction was conducted in 96-well plates, and NADPH oxidation was monitored by measuring OD at 340 nm over 5 min at 25 °C using a FLUOstar Omega plate reader.

### OXPHOS assay (Seahorse)

MIAPaCa-2 cells were plated at 100,000 cells/well in a 24-well plate (Seahorse) and incubated overnight. Then, cells were treated with 6 µM sorafenib and/or 6 µM LZX-2-73 for 6 h. The Oxygen Consumption Rate (OCR) (pmol O_2_/min) was measured using the Seahorse Bioscience XF24 Extracellular Flux Analyzer (Agilent) in response to 1 μM oligomycin, 0.25 μM carbonylcyanide p-(trifluoro-methoxy) phenylhydrazone (FCCP), 0.5 µM rotenone/Antimycine A (Millipore Sigma).

### Catalase and SOD activity assay

MIAPaCa-2 cells were treated with 6 µM sorafenib and/or 6 µM LZX-2-73 for 24 h. Then, cells were collected and lysed in PBS with a sonicator. For the determination of catalase or SOD activity, we employed the Catalase (CAT) Activity Assay Kit (E-BC-K031-M) and Total Superoxide Dismutase (T-SOD) Activity Assay Kit (WST-1 Method) (E-BC-K020-M), respectively. For the determination, we followed the manufacturer’s instructions. For both kits, the absorbance was measured in a FLUOstar Omega microplate reader (BMG Labtech). For the normalization, protein level was determined by Pierce™ BCA Protein Assay (Thermo Fisher).

### Western blotting

MiaPaCa-2 cells were treated with 6 µM sorafenib and/or 6 µM LZX-2-73 for 24 or 48 h. Then, cells were collected and lysed in RIPA buffer (0.5 M Tris-HCl, pH 7.4, 1.5 M NaCl, 2.5% deoxycholic acid, 10% NP-40, 10 mM EDTA). Then, 25 µg of each protein lysate was loaded in an acrylamide gel and transferred to a nitrocellulose membrane. The membranes were incubated with the primary antibody overnight at 4 °C and with an IgG-HRP conjugated secondary antibody for 1 h. For detection, we use the reagent ECL detection system (Millipore Corp., Bedford). The primary antibodies used in this study were: Survivin (1:1000, Cell Signaling 2808), NUPR1 (1:1000, homemade), B-RAF (1:1000, Cell Signaling 14814), phospho-B-RAF (1:1000, Cell Signaling 2696), NRF2 (Proteintech 16396-1-AP), NRF2 (phospho Ser40) (1:1000, ab76026), GPX4 (1:1000, Abcam 125066) and xCT/SLC7A11 (D2M7A) (1:1000, Cell Signaling 12691) and beta-Actin (1:15,000, Proteintech 81115-1-RR).

### RT-qPCR

Total RNA was extracted from cells using the RNeasy kit (Qiagen), and cDNA was obtained by reverse transcription using the Go Script kit (Promega), according to the manufacturer’s instructions. Real-time quantitative PCR (RT-qPCR) was performed using the AriaMx system (Agilent). Primer sequences are listed below: Nupr1-F:5′-TAGAGACGGGACTGCG-3′; Nupr1-R: 5′-GCGTGTCTATTTATTGTTGC-3′; 36B4-F:5′-AATCCCTGACGCACCGCCGTGATG-3′; 36B4-R:5′-TGGGTTGTTTTCCAGGTGCCCTCG-3′.

### ROS and mROS determination

Cells were seeded at 50,000 cells per well in 12-well plates, and treated the next day as indicated. After 24 h of treatment, the cells were detached with trypsin, collected, and rinsed with PBS. Subsequently, CellROX Green at 5 µM (Thermo Fisher C10444) or Mitosox Red at 5 µM (Thermo Fisher M36008) diluted in HBSS were added and incubated for 30 min at 37 °C + 5% CO_2_ in the dark. Excess probe was removed by washing the cells with HBSS, and Hoechst (1:2000) was added to stain dead cells. Fluorescence signals were measured using a flow cytometer (Cytoflex S). A minimum of 10,000 cells was analyzed for each condition. Data were analyzed using FlowJO software on median fluorescence level gated on live cells.

### Xenografts development

Female Crl:NU(Ico)-*Foxn1*^*nu*^ mice, aged 4 weeks, were obtained from Charles River Company. The mice were housed in the Experimental Animal House at the Centre de Cancérologie de Marseille, pôle Luminy, under specific pathogen-free conditions. All procedures followed laboratory animal care guidelines and ethical regulations. At 5 weeks old, the mice were subcutaneously inoculated with 10 million MIAPaCa-2 cells mixed with 50 µL of Matrigel (BD Pharmingen). When tumors reached a size of 200 mm³, the mice received daily intraperitoneal injections of either 5% DMSO in sunflower seed oil (vehicle), 25 mg/kg sorafenib, 10 mg/kg LZX-2-73, or a combination of 25 mg/kg sorafenib and 10 mg/kg LZX-2-73. The mice were weighed and tumor volumes measured with calipers twice a week, with tumor volumes calculated assuming an ellipsoid shape. After 28 days of treatment, the mice were sacrificed.

### Hematoxylin and eosin staining

Serial 4 µm sections were cut from each paraffin-embedded tissue sample using a Leica Histocore Biocut (Leica, Germany). These sections were then stained with hematoxylin and eosin according to the manufacturer’s instructions using Leica Autostainer XL (Leica, Germany). Photomicrographs were captured using a ZEISS Axio Imager Z2 microscope (Zeiss, Germany).

### In situ detection of apoptotic cells in tumor tissue

Serial 4 µm sections were prepared from each paraffin-embedded tumor sample using a Leica Histocore Biocut (Leica, Germany). Apoptotic cells were analyzed using the TUNEL Assay Kit - HRP-DAB (ab206386, Abcam) following the manufacturer’s protocol. Images of the sections were obtained with a ZEISS Axio Imager Z2 microscope (Zeiss, Germany).

### Immunohistochemistry

Four-micrometer paraffin sections of tumors were incubated for half an hour at 65 °C and then rehydrated. Antigen retrieval was performed by incubating the slides in Citrate Buffer TRS pH 6 (Dako) at 96 °C for 20 min, followed by a 30-min incubation at room temperature to cool them down. Endogenous peroxidases were blocked using 3% H_2_O_2_ for 10 min, and the slides were rinsed three times with PBS. The slides were then incubated with monoclonal rabbit Anti-Human Ki-67 antibody (Abcam, 1:100) and rabbit polyclonal anti-4-Hydroxynonenal antibody (Abcam, 1:200) for 60 min at room temperature. After three washes in PBS, the slides were incubated with a biotinylated Goat anti-Rabbit Ig secondary antibody (Abcam, 1:400) for 30 min at room temperature. Following another three PBS washes, Streptavidin-HRP (Agilent; 1:500) was added and incubated for 30 min at room temperature. The staining was developed using diaminobenzidine (DAKO, Agilent) for 10 min at room temperature. The slides were rinsed in distilled water, counterstained with Mayer’s Hematoxylin for 30 s, and blued with 0.1% sodium bicarbonate solution for 3 min. Finally, the slides were dehydrated, cleared, and mounted with coverslips and permanent mounting medium. Images of the sections were captured using a ZEISS Axio Imager Z2 microscope (Zeiss, Germany).

### Statistics

Statistical analyses were performed using one-way ANOVA, Dunnett’s test, or two-way ANOVA, Dunnett’s test. Results were presented as the mean ± SD from at least three independent experiments. A *p*-value of <0.05 was considered statistically significant.

## Results

### Evaluation of the synergistic anticancer activity of sorafenib and LZX-2-73

**Fig. 1:**
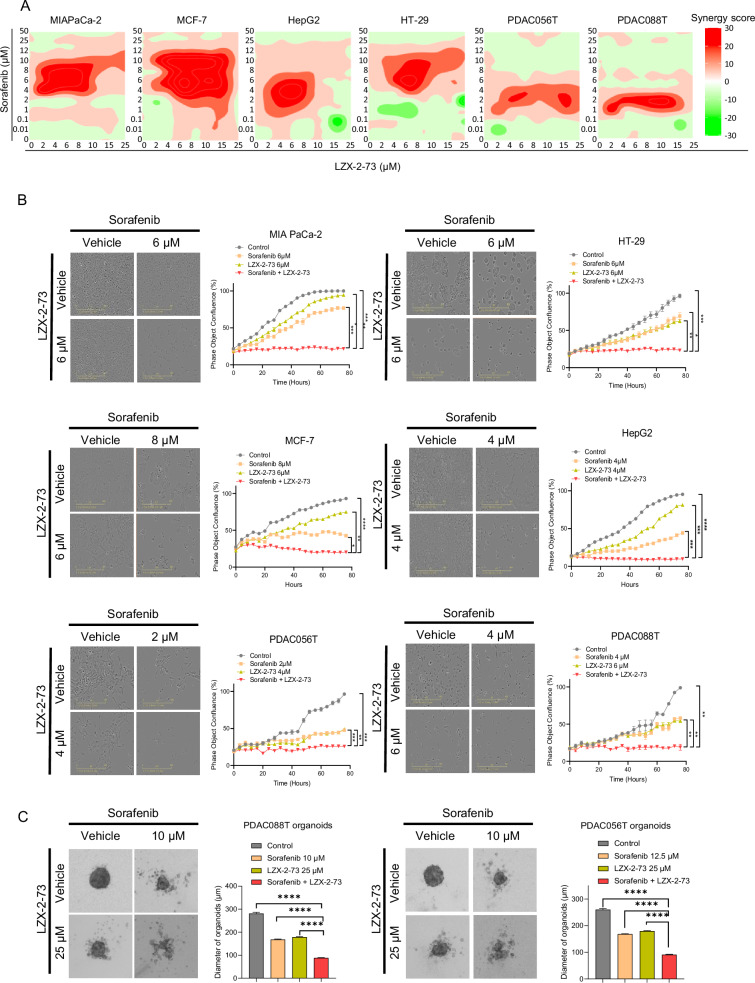
LZX-2-73 and sorafenib combination showed a synergistic anticancer effect. **A** HSA synergy score distribution plots for the combination treatment of LZX-2-73 with sorafenib. **B** Representative images after 72 h of treatment with sorafenib, LZX-2-73, or their combination to MIAPaCa-2, HT-29, MCF-7, HepG2, PDAC056T, or PDAC088T cells (left) and the quantification of cell confluency in the images (right) by the Incucyte device. Data are shown as mean ± SEM. *p < 0.05, **p < 0.01, ***p < 0.001, ****p < 0.0001 (2-way ANOVA, Dunnett’s test) (*n* = 3), statistic shown on the last point of measurement, compared to combinatory treatment. **C** Representative images upon 72 h of treatment with sorafenib, LZX-2-73, or their combination to PDAC056T organoids and PDAC088T organoids (left) and the quantification of organoid diameters in the images (right). Data are shown as mean ± SEM. **p < 0.01, ***p < 0.001, ****p < 0.0001 (1-way ANOVA, Dunnett’s test, compared to combinatory treatment) (*n* = 3).

In this study, we assessed the anticancer efficacy of various clinically used chemotherapeutic agents -SN38, oxaliplatin, gemcitabine, paclitaxel, and 5-FU- along with the targeted drug sorafenib, in combination with the novel NUPR1 inhibitor LZX-2-73. This evaluation was performed across 15 cancer cell lines (both commercial and primary) using a dose-response matrix. The SynergyFinder tool was employed to calculate synergy scores for each drug combination (Table 1). Combinations such as paclitaxel and oxaliplatin with LZX-2-73 yielded synergy scores between −10 and 10, indicating predominantly additive effects. Notably, higher synergy scores were observed in cells with greater IC50 values upon treatment, particularly with sorafenib, suggesting that the combination could significantly enhance therapeutic efficacy. Among all tested agents, sorafenib demonstrated the most consistent and pronounced synergy across all cell lines. Consequently, we focused on further investigating the combinatory antitumoral effects of sorafenib and LZX-2-73. As shown in Fig. 1A, the combination exhibited strong synergistic activity in all tested cells within the dose-response matrices.

**Table 1:** Synergy score of LZX-2-73 combination with several chemotherapeutic agents.

Cancer cell line	Synergy score between LZX-2-73 and chemotherapeutic agents					
	SN38	Oxaliplatin	Gemcitabine	Paclitaxel	5-FU	Sorafenib
MIAPaCa-2	24.5	4.1	10.8	19.6	−0.3	23.2
PDAC056T	4.8	1.0	−1.9	−4.0	3.4	12.2
PDAC079T	4.8	−1.5	1.3	4.0	1.5	8.4
PDAC088T	5.0	0.7	−5.8	−2.9	3.0	16.2
PDAC018T	16.5	−2.8	4.1	−3.0	3.6	10.8
PC-3	−1.4	−4.1	0	−10.5	6.5	16.8
HT-29	24.2	−1.7	3.2	16.9	−4.9	17.6
H358	19.7	−0.3	1.8	14.3	5.3	18.6
H1299	14.4	−4.2	13.9	20.4	−1.9	22.3
MCF-7	33.6	2.4	10.8	6.2	16.8	42.1
MDA-MB-231	17.3	5.9	4.6	15.3	4.0	19.0
Jurkat	2.4	−2.2	−5.2	−3.7	0.8	11.4
Daudi	1.0	−10.7	−1.6	−2.4	−5.4	−4.7
U87	8.8	−2.5	1.0	7.0	−6.6	14.7
HepG2	8.4	3.5	4.1	6.2	6.0	17.3
Hep3B	20.2	0.3	4.9	23.7	4.3	13.9

Different cell lines and patient-derived primary cultures were co-treated with the NUPR1 inhibitor LZX-2-73 and SN38, oxaliplatin, gemcitabine, paclitaxel, 5-FU, and sorafenib for 72 h. Cell viability was determined using SynergyFinder software. The Synergy score was determined according to the HSA model. A synergy score below −10 indicated antagonistic interactions, scores between −10 and 10 indicated additive interactions, and scores above 10 indicated synergistic interactions.

For further analysis, we selected six cancer cell lines, MIAPaCa-2, HT-29, MCF-7, HepG2, PDAC056T, and PDAC088T- based on their highest synergy scores. Drug concentrations were predefined to optimize the synergistic interaction between sorafenib and LZX-2-73. To evaluate the impact on cell proliferation, we monitored cell confluency in real-time using the Incucyte device, comparing treatments with sorafenib alone, LZX-2-73 alone, and their combination. As shown in Fig. 1B, the combination treatment significantly suppressed cell proliferation in all six cancer cell lines compared to individual treatments, highlighting a potent synergistic effect. Additionally, Fig. 1C illustrates that the sorafenib and LZX-2-73 combination markedly reduced the diameter of primary PDAC organoids, demonstrating a strong inhibitory effect on organoid formation. In conclusion, our findings indicate that the combination of sorafenib and LZX-2-73 consistently outperforms conventional chemotherapeutic agents, exhibiting robust synergistic effects across all tested cancer models.

### The combination of sorafenib and LZX-2-73 enhances cancer cell death

**Fig. 2:**
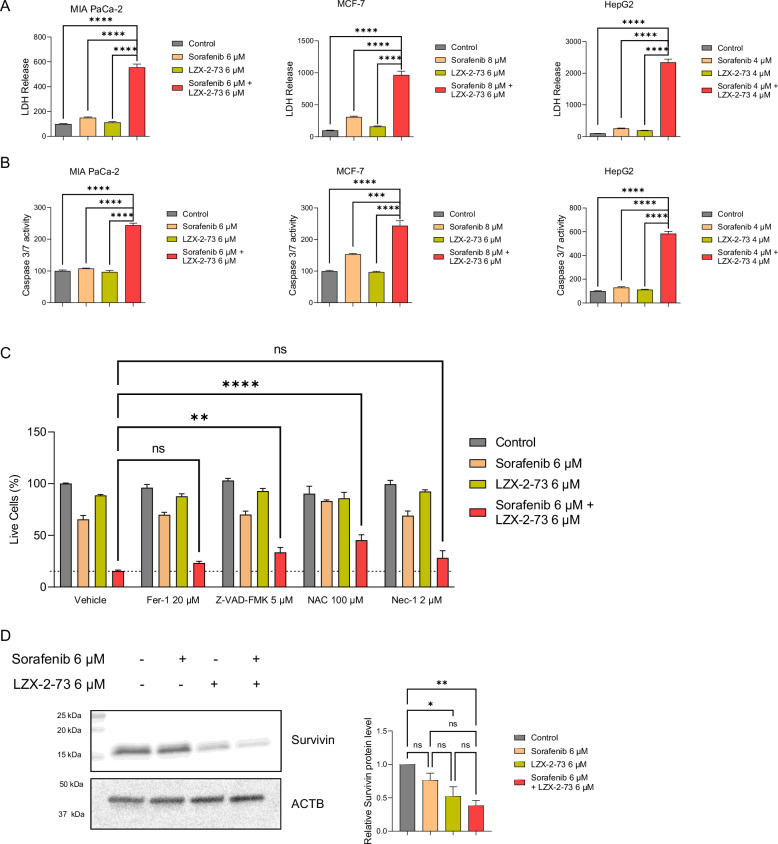
Combination of sorafenib and LZX-2-73 resulted in increased cell death. **A** LDH release and **B** caspase 3/7 activity were measured on MIAPaCa-2 cells, MCF-7 cells, and HepG2 cells, respectively. The cells were treated with single drugs and the drug combination for 48 h. Data are shown as mean ± SEM. Statistical significance is indicated as follows: *p < 0.05, **p < 0.01, ***p < 0.001, ****p < 0.0001 (1-way ANOVA, Dunnett’s test, compared to combinatory treatment) (*n* = 3). **C** Viability of MIAPaCa-2 cells upon a 72-h treatment with sorafenib, LZX-2-73, or their combination, in the presence or not of several cell death inhibitors. **D** Expression of survivin, analyzed by western blot, in response to treatment with LZX-2-73 alone or in combination with sorafenib. Data are shown as mean ± SEM. Statistical significance is indicated as follows: *p < 0.05, **p < 0.01, ***p < 0.001, ****p < 0.0001 (1-way ANOVA, Dunnett’s test, compared combinatory treatment) (*n* = 4).

Building on our findings that the combination of sorafenib and LZX-2-73 exerts strong synergistic antitumor effects across multiple cancer types, we investigated the underlying mechanisms of cell death induced by this treatment. To achieve this, we measured lactate dehydrogenase (LDH) release and caspase 3/7 activity at various time points. Our results revealed that the combination treatment significantly increased LDH release compared to single-drug treatments across all tested cell lines (Fig. 2A, Supplementary Fig. 2A), indicating heightened membrane damage and cell death. Furthermore, caspase 3/7 activity was markedly elevated in the combination group, surpassing levels observed in the individual drug treatments (Fig. 2B, Supplementary Fig. 2B), suggesting enhanced apoptotic cell death. To gain deeper insight into the specific cell death mechanisms involved, we utilized various inhibitors, including Nec-1 (necroptosis inhibitor), Z-VAD-FMK (apoptosis inhibitor), Fer-1 (ferroptosis inhibitor), and NAC (antioxidant). Interestingly, treatment with Z-VAD-FMK and NAC partially rescued cell viability, indicating that the combinatory treatment induces both apoptosis and oxidative stress-driven cell death. These findings suggest that the anticancer activity of sorafenib and LZX-2-73 is mediated through the dual activation of apoptotic pathways and oxidative stress (Fig. 2C).

### Regulation of survivin expression by LZX-2-73 and sorafenib

Survivin, a member of the inhibitor of apoptosis (IAP) protein family, plays a central role in tumor cell survival by suppressing apoptosis and regulating cell cycle progression [45]. Its aberrant expression has been associated with poor prognosis and therapy resistance in several cancers [46]. Previous studies have shown that NUPR1 can transcriptionally regulate genes involved in apoptosis and stress responses, including survivin, thereby promoting cancer cell survival [47]. In addition, Sorafenib has been reported to modulate survivin expression and reduce its levels under certain conditions [48]. These findings provide a biological rationale for investigating survivin expression in the context of NUPR1 inhibition and Sorafenib treatment.

To investigate this, we evaluated protein levels under different treatment conditions. Cells treated with Sorafenib exhibited a modest reduction in survivin expression compared to untreated controls, suggesting a limited effect of this multi-kinase inhibitor on anti-apoptotic signaling pathways involving survivin. In contrast, treatment with LZX-2-73, a selective inhibitor of NUPR1, led to a significantly greater decrease in survivin levels, indicating that NUPR1 activity contributes more directly to the regulation of this anti-apoptotic factor. Interestingly, combined treatment with LZX-2-73 and Sorafenib resulted in the most pronounced suppression of survivin expression observed among all conditions (Fig. 2D). These results support the hypothesis that NUPR1 inhibition not only promotes ferroptotic cell death, as previously reported [37], but also contributes to apoptotic signaling pathways. The downregulation of survivin likely plays a key role in this process, positioning survivin as a molecular link between ferroptosis and apoptosis in the context of NUPR1-targeted therapy.

### Sorafenib and LZX-2-73 induce massive oxidative stress in tumor cells

**Fig. 3:**
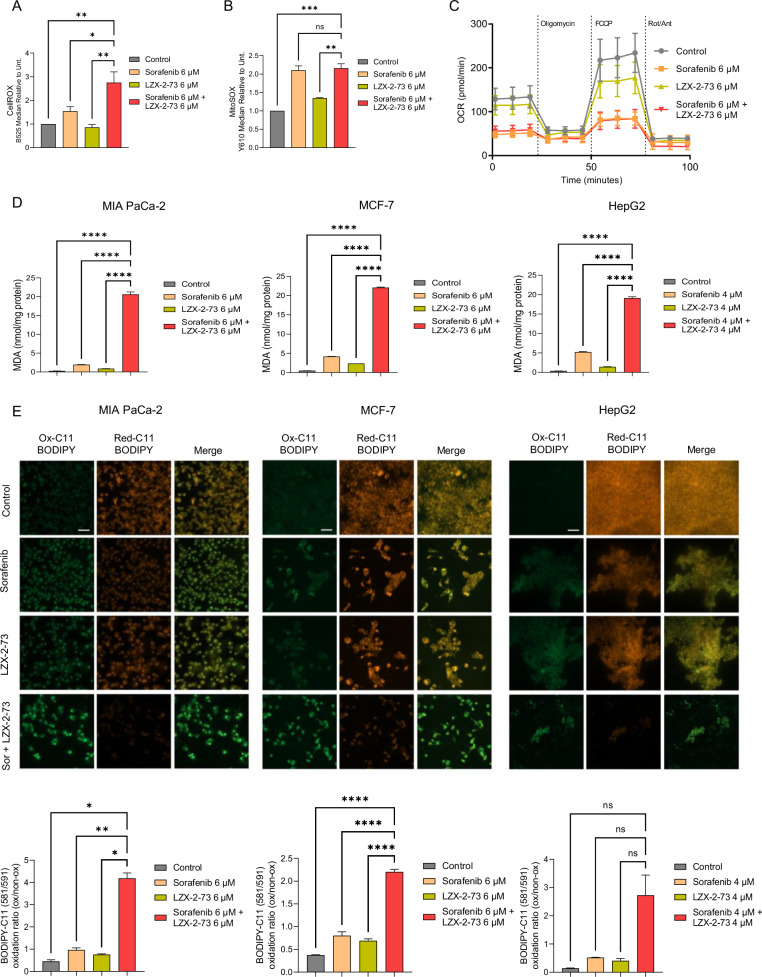
Combination of sorafenib and LZX-2-73 led to an increase in ROS accumulation. ROS or mitochondrial ROS production was detected using CellROX (**A**) or MitoSOX (**B**), respectively, by flow cytometry analysis on MIAPaCa-2 cells incubated for 24 h with sorafenib, LZX-2-73, or their combination. Statistical significance is indicated as follows: *p < 0.05, **p < 0.01, ***p < 0.001 (1-way ANOVA, Dunnett’s test, compared to combinatory treatment). (*n* = 3). **C** OXPHOS metabolism, reflected by oxygen consumption rate (OCR) levels were measured on MIAPaCa-2 cells incubated for 24 h with sorafenib, LZX-2-73, or their combination Levels of lipid peroxidation, indicated by MDA levels (**D**) or by the oxidation of the BODIPY-C11 probe (**E**) (measured via fluorescence microscopy to monitor the oxidized and non-oxidized variants of lipid peroxides), were assessed in cells treated with sorafenib, LZX-2-73, or the combination of both drugs for 72 h. Statistical significance is indicated as follows: *p < 0.05, **p < 0.01, ****p < 0.0001 (1-way ANOVA, Dunnett’s test, compared to combinatory treatment) (*n* = 3). Scale bar: 50 μm.

Given our previous findings that antioxidants partially rescued cells from death following sorafenib and LZX-2-73 co-treatment, we further examined whether this combination therapy leads to reactive oxygen species (ROS) accumulation and its subsequent effects. To assess ROS levels, we measured intracellular ROS upon treatment. As shown in Fig. 3A, the combination treatment induced a substantial accumulation of ROS compared to sorafenib alone. To determine whether mitochondria were the primary source of ROS, we measured mitochondrial ROS levels. Although co-treatment resulted in a higher increase in mitochondrial ROS than the control, the levels were not significantly greater than those induced by sorafenib alone (Fig. 3B). This observation aligns with the fact that sorafenib itself causes considerable mitochondrial damage, as measured by the Seahorse assay (Fig. 3C), which was not further exacerbated by LZX-2-73. Cellular lipids, particularly those in the plasma membrane, are highly susceptible to peroxidation. To evaluate lipid peroxidation, we quantified malondialdehyde (MDA), a key byproduct of lipid oxidation. Compared to single-drug treatments, the combination therapy led to a significant increase in MDA accumulation across all tested cell lines (Fig. 3D, Supplementary Fig. 2A). Additionally, fluorescence microscopy using BODIPY^TM^ 581/591 C11 staining confirmed a substantial rise in lipid hydroperoxides, along with increased levels of oxidized lipid peroxides in the combination treatment group compared to either drug alone (Fig. 3E, Supplementary Fig. 2B). Together, these findings demonstrate that the sorafenib and LZX-2-73 combination induces a pronounced accumulation of ROS, which in turn leads to extensive lipid peroxidation, contributing to the synergistic cell death observed in cancer cells.

### Co-treatment with sorafenib and LZX-2-73 suppresses the oxidative stress response in tumor cells

**Fig. 4:**
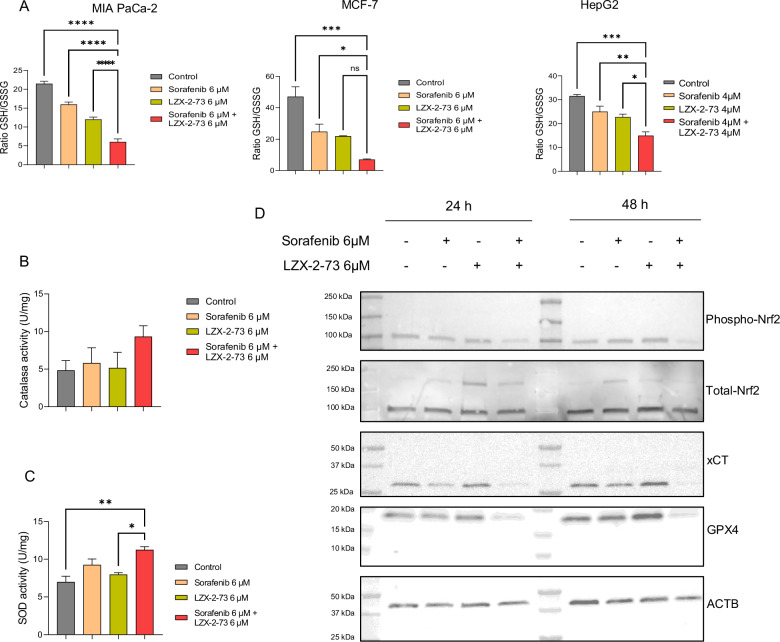
Combination of sorafenib and LZX-2-73 depleted the antioxidative stress response in tumor cells. **A** The ratio of GSH/GSSG was measured in MIAPaCa-2 cells, MCF-7 cells, and HepG2 cells treated with sorafenib, LZX-2-73, or the combination of the two drugs for 72 h. **B** Catalase and **C** SOD activity was measured in MIAPaCa-2 cells treated with sorafenib, LZX-2-7,3, or the combination of the two drugs for 24 h. **D** Western blot of phospho-NRF2, total NRF2, XCT, GPX4, and actin was performed in MIAPaCa-2 cells treated with sorafenib, LZX-2-7,3, or the combination of the two drugs for 24 or 48 h.

Excessive ROS accumulation can trigger an oxidative stress response, activating transcription factors and mobilizing the cellular antioxidant defense system, including enzymatic and non-enzymatic antioxidants. Given the significant ROS buildup observed with sorafenib and LZX-2-73 co-treatment, we investigated its impact on the antioxidant response. The ratio of reduced glutathione (GSH) to oxidized glutathione disulfide (GSSG) is a critical determinant of a cell’s susceptibility to oxidative stress. To assess whether the combination treatment disrupts glutathione homeostasis, we measured intracellular GSSG levels and calculated the GSH/GSSG ratio. As shown in Fig. 4A and Supplementary Fig. 3A, co-treatment significantly reduced the GSH/GSSG ratio while markedly increasing GSSG levels across all tested cell models, indicating substantial reduced glutathione depletion and thus acute oxidative stress. Next, we examined the activity of key antioxidant enzymes. Catalase, a major ROS-detoxifying enzyme, was not significantly activated by the combination treatment (Fig. 4B), whereas superoxide dismutase (SOD) activity increased upon co-treatment (Fig. 4C), suggesting a partial compensatory response. To further investigate the oxidative stress response, we analyzed NRF2, the master regulator of antioxidant defense. Notably, phosphorylated NRF2 (p-NRF2 Ser40) levels were reduced following co-treatment (Fig. 4D), suggesting impaired NRF2 activation. Consistently, the expression of key NRF2 target proteins, including XCT (the functional cystine-glutamate antiporter involved in glutathione synthesis) and GPX4 (a crucial phospholipid hydroperoxidase that protects against lipid peroxidation and uses GSH as a substrate), was significantly downregulated at 24- and 48-h post-treatment (Fig. 4D). Collectively, these results demonstrate that sorafenib and LZX-2-73 co-treatment suppresses the oxidative stress response by depleting glutathione levels and downregulating essential antioxidant transcription factors and detoxification proteins.

### Synergistic antitumor effects of sorafenib and LZX-2-73 in a PDAC xenograft model

**Fig. 5:**
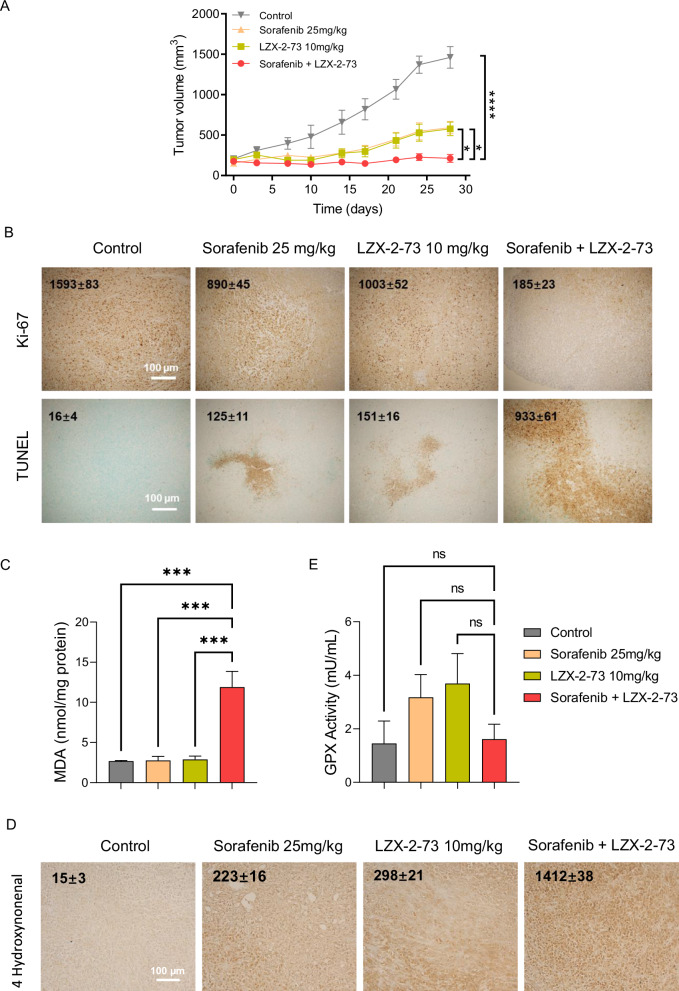
Synergistic antitumor effect of combined sorafenib and LZX-2-73 treatment in a tumor xenograft mouse model. Female Crl:NU(Ico)-*Foxn1*^*nu*^ mice implanted with pancreatic cancer MIAPaCa-2 cell lines xenografts and treated daily with sorafenib (25 mg/kg), LZX-2-73 (10 mg/kg), or a combination of both drugs (25 mg/kg + 10 mg/kg) via intraperitoneal injection. **A** Tumor volume was measured twice per week. Data are shown as mean ± SEM. *p < 0.05, ****p < 0.0001 (1-way ANOVA, Dunnett’s test, compared to combinatory treatment). **B** Representative images of tissue sections following Ki-67 IHC staining and TUNEL assay from mice treated with sorafenib (25 mg/kg), LZX-2-73 (10 mg/kg), or their combination. Scale bar: 100 μm. **C** The levels of malondialdehyde (MDA) and (**D**) 4-Hydroxynonenal, and (**E**) the activity of glutathione peroxidase (GPx) were assessed in MIAPaCa-2 xenografted tumors following a 4-week treatment with sorafenib, LZX-2-73, or a combination of both. Statistical significance is indicated as follows: ns: not significant, ***p < 0.001 (1-way ANOVA, Dunnett’s test, compared to combinatory treatment). Scale bar: 100 μm.

To further evaluate the synergistic antitumor effects of sorafenib and LZX-2-73 in vivo, we established a subcutaneous xenograft model by injecting MIAPaCa-2 cells into nude mice. As shown in Fig. 5A, monotherapy with either sorafenib or LZX-2-73 moderately inhibited tumor growth, whereas combination treatment resulted in near-complete suppression of tumor progression. To assess the impact on tumor proliferation, we performed immunohistochemical (IHC) analysis using an anti-Ki-67 antibody, a widely used proliferation marker. The results revealed a significant reduction in Ki-67-positive tumor cells in the combination group compared to the monotherapy groups (Fig. 5B), indicating a strong antiproliferative effect. Additionally, TUNEL staining confirmed a markedly higher incidence of apoptosis in tumors treated with the combination therapy compared to single-drug treatments (Fig. 5B). Given the role of oxidative stress in the observed tumor suppression, we measured lipid peroxidation markers in tumor tissues. Combination treatment significantly increased malondialdehyde (MDA) levels compared to monotherapy (Fig. 5C) and also elevated 4-hydroxynonenal (4-HNE) levels, two by-products of lipid peroxidation (Fig. 5D), indicating substantial lipid oxidative damage. Notably, although both sorafenib and LZX-2-73 individually induced a slight increase in glutathione peroxidase (GPx) activity, the combination treatment did not further enhance GPx activity (Fig. 5E), aligning with our in vitro findings of an impaired oxidative stress response that seems mainly due to a perturbation of the XCT/GSH/GPX4 axis. Importantly, no adverse effects were observed in treated mice throughout the experiment. All mice exhibited a slight but steady increase in body weight, with no significant differences between treatment groups (Supplementary Fig. 4A). Histological analysis of major organs (heart, liver, spleen, lungs, and kidneys) via hematoxylin and eosin (H&E) staining (Supplementary Fig. 4B) showed no evidence of tissue damage or pathological changes, indicating that the combination therapy was well tolerated. Overall, these findings provide strong in vivo evidence that the combination of sorafenib and LZX-2-73 exerts a potent synergistic anticancer effect, significantly inhibiting tumor growth through oxidative stress induction and apoptosis, while remaining non-toxic to healthy tissues.

### Effect of LZX-2-73 and sorafenib on NUPR1 expression in MIAPaCa-2 cells

To evaluate the impact of sorafenib and LZX-2-73 on NUPR1 expression, we treated MIAPaCa-2 cells with sorafenib alone, LZX-2-73 alone, or a combination of both compounds. NUPR1 mRNA expression was assessed by RT-qPCR, and protein levels were analyzed by Western blotting. Data is presented in Supplementary Fig. 5A, B. Treatment with LZX-2-73 alone led to a significant upregulation of NUPR1 mRNA compared to control cells. This transcriptional activation was also maintained when LZX-2-73 was combined with sorafenib. In contrast, treatment with sorafenib alone had a minimal effect on NUPR1 mRNA levels. At the protein level, a modest increase in NUPR1 expression was observed following LZX-2-73 treatment, consistent with the transcriptional findings, although the magnitude of change was less pronounced. The combined treatment resulted in a slightly higher protein signal compared to either agent alone, but the difference did not reach statistical significance. These results indicate that LZX-2-73 is capable of inducing NUPR1 expression at the transcriptional level. This supports a potential role for LZX-2-73 in modulating stress-response pathways involving NUPR1 in pancreatic cancer cells.

We also investigated whether the combination of LZX-2-73 with sorafenib enhances the effect of sorafenib as a multi-kinase inhibitor. To this end, we evaluated a well-characterized target of sorafenib: the phosphorylation of B-RAF. MIAPaCa-2 cells were treated with sorafenib alone or in combination with LZX-2-73, and we observed that the presence of the NUPR1 inhibitor does not alter sorafenib’s activity on phosphorylatable substrates (Supplementary Fig. 5C). In conclusion, the synergistic antitumor effect observed between both compounds does not appear to result from a modification of sorafenib’s kinase-inhibitory capacity.

## Discussion

Sorafenib is an oral multi-kinase inhibitor that targets tumor angiogenesis and reduces the nutrient supply to tumor cells, thereby inhibiting tumor proliferation and metastasis [49, 50]. In preclinical studies, sorafenib has demonstrated the ability to inhibit the growth of various human xenograft tumors, including renal cell carcinoma, ovarian cancer, PDAC, colon cancer, thyroid cancer, breast cancer, and melanoma [51–54]. The FDA has approved sorafenib as an oral anticancer medication for the treatment of HCC, renal cell carcinoma, and thyroid cancer [55]. While sorafenib is primarily approved as monotherapy for these cancers, ongoing clinical trials are evaluating its efficacy in combination with other drugs. Most of these trials are in phases I or II, with a predominant focus on HCC. Some trials have reported promising results, benefiting cancer patients; however, further research is necessary due to limited sample sizes [56, 57]. To date, no sorafenib combination regimen has received FDA approval, highlighting the need for the development of more effective combination therapies.

Recent research on sorafenib as an oxidative stress inducer in combination therapy has primarily focused on increasing the sensitivity of HCC to sorafenib. For example, the fatty acid synthase (FASN) inhibitor orlistat exhibits significant synergistic anti-HCC effects with sorafenib and can reverse sorafenib resistance. This effect is attributed to the solute carrier family 7 member 11 (SLC7A11, also called xCT System), which plays a critical role in sorafenib resistance. The downregulation of FASN activates ferroptosis triggered by SLC7A11, thereby overcoming sorafenib resistance [58]. Additionally, tiliroside, a natural flavonoid glycoside isolated from the oriental paperbush flower, has demonstrated synergistic anticancer activity in combination with sorafenib, enhancing HCC sensitivity to sorafenib. Tiliroside directly binds to and inhibits TANK-binding kinase 1 (TBK1), thereby suppressing the phosphorylation of serine 349 on sequestosome-1 (p62). This inhibition decreases the affinity of p62 for kelch-like ECH-associated protein 1 (Keap1), promoting the ubiquitination and degradation of nuclear factor erythroid 2-related factor 2 (Nrf2). Consequently, ferroptosis is induced, enhancing the sensitivity of HCC cells to sorafenib [59]. Nrf2 upregulation-induced ferroptosis resistance weakens the anticancer efficacy of sorafenib [60–62]. To address this issue, researchers have employed “click” chemistry-based multifunctional hyperbranched nanocarriers for the co-delivery of sorafenib and siNrf2. This approach overcomes Nrf2-mediated ferroptosis resistance and enhances sorafenib’s antitumor effects [63]. Interestingly, our results indicate that the combination of sorafenib and LZX-2-73 induces massive oxidative stress while reducing NRF2 activation. This suppression of NRF2 prevents the oxidative stress response, ultimately promoting cell death. Although sorafenib can induce ferroptosis in some models, it fails to do so across a wide range of models [64], suggesting diverse mechanisms of action primarily involving oxidative stress. Notably, when ferroptosis was inhibited in co-treated cells with LZX-2-73, Fer-1 failed to rescue cell viability, whereas antioxidants such as NAC successfully prevented cell death. Furthermore, sorafenib negatively impacts mitochondrial activity through oxidative and nitrosative stress [65], consistent with our observations. Sorafenib monotherapy already induces significant mitochondrial damage, which is not further exacerbated by co-treatment with LZX-2-73. Additionally, essential oxidative stress-response proteins, including xCT and GPX4, were downregulated upon combinatory treatment, implicating a severe and unresolved oxidative stress response that drives cell death through ROS accumulation.

NUPR1, a nuclear transcription factor, plays a crucial role in regulating the oxidative stress response [66]. For instance, Liu et al. identified NUPR1 as a key inhibitor of ferroptosis, demonstrating that it transcriptionally activates lipocalin-2 (LCN2), which significantly mitigates oxidative damage and suppresses ferroptosis in pancreatic cancer cells [67, 68]. Similarly, Zhang et al. showed that circPIAS1 confers resistance to ferroptosis in HCC cells by upregulating NUPR1. Moreover, Huang et al. found that the NUPR1 inhibitor ZZW-115 downregulates mitochondrial biogenesis regulator Mitochondrial Transcription Factor A (TFAM), thereby reducing the antioxidant capacity in pancreatic cancer cells [66]. Massive oxidative stress induction, including ferroptosis, leads to cell death, characterized by ROS accumulation, lipid peroxidation, decreased antioxidative enzyme activity (GPX, SOD, catalase), reduced cystine/glutamate antiporter (system xc-) function, and glutathione (GSH) depletion [13, 69, 70]. Our findings align with this phenomenon, as the combinatory treatment led to a significant disruption of the oxidative stress response. These results suggest that combining the NUPR1 inhibitor LZX-2-73 with sorafenib could yield synergistic anticancer effects via oxidative stress induction.

We demonstrated that NUPR1 is a promising target for the treatment of different types of cancers. However, due to the properties of this protein, the design of a clinically applicable pharmacological inhibitor has been a challenge. The most potent and best-studied inhibitor to date against NUPR1, the compound ZZW-115, has been delayed for clinical use due to its ability to interact with hERG receptors, a classic problem in the pharmaceutical industry [71]. This new NUPR1 inhibitor, LZX-2-73, has shown strong antitumor activity and no affinity for hERG receptors [72], making it a potentially safer drug agent for use in humans.

Although sorafenib can induce ferroptosis in certain cellular contexts, this effect is not consistent across all models, likely due to the activation of antioxidant defense mechanisms such as the NUPR1/LCN2 and NUPR1/NRF2 pathways. In our study, we observed that the combination of sorafenib with the NUPR1 inhibitor LZX-2-73 amplifies oxidative stress while disrupting these protective responses. Interestingly, although the molecular profile of co-treated cells suggests a ferroptosis-prone environment, the lack of rescue by the ferroptosis inhibitor Fer-1 indicates that cell death proceeds through a ferroptosis-independent, ROS-mediated mechanism. This suggests that targeting NUPR1 can sensitize cancer cells to oxidative damage induced by sorafenib, broadening its therapeutic potential. The central finding of this research underscores the potential of rational drug combinations to provide significant therapeutic benefits, particularly for patients with limited treatment options. Our study reveals that LZX-2-73 not only enhances sorafenib’s efficacy through synergistic interaction but also transforms sorafenib-resistant cancer cells into responsive ones. This discovery presents a promising therapeutic strategy for overcoming drug resistance in cancer treatment, a longstanding challenge in medical research.

In conclusion, our study demonstrates that combining the multi-target kinase inhibitor sorafenib with the novel, safe NUPR1 inhibitor LZX-2-73 produces significantly stronger synergistic effects than other chemotherapeutic agents. This potent combination therapy not only exhibits superior synergy in liver cancer but also extends its efficacy to pancreatic, prostate, colorectal, non-small cell lung cancer, breast cancer, leukemia, and glioblastoma. Our findings highlight that the sorafenib and LZX-2-73 combination markedly enhances cancer cell death, effectively inhibits tumor growth, boosts anticancer activity in vivo, and allows for a reduced monotherapy dosage. We believe this research could broaden sorafenib’s clinical application, expanding treatment options for cancer patients and offering new therapeutic alternatives for those in need.

## Supplementary information

Supp Figure 1

Supp Figure 2

Supp Figure 3

Supp Figure 4

Supp Figure 5

Legend of Supplementary Figures

Original blots uncropped

## Data Availability

Data are available upon request.
