# Supplementary figures and images for: Synergistic antitumor activity of sorafenib and the NUPR1 inhibitor LZX-2-73 in multiple cancer models
Source: Cell Death Dis. 2025 Nov 17;16(1):839. doi: 10.1038/s41419-025-08178-8 (PMC12623841; doi:10.1038/s41419-025-08178-8)

## Slide 1
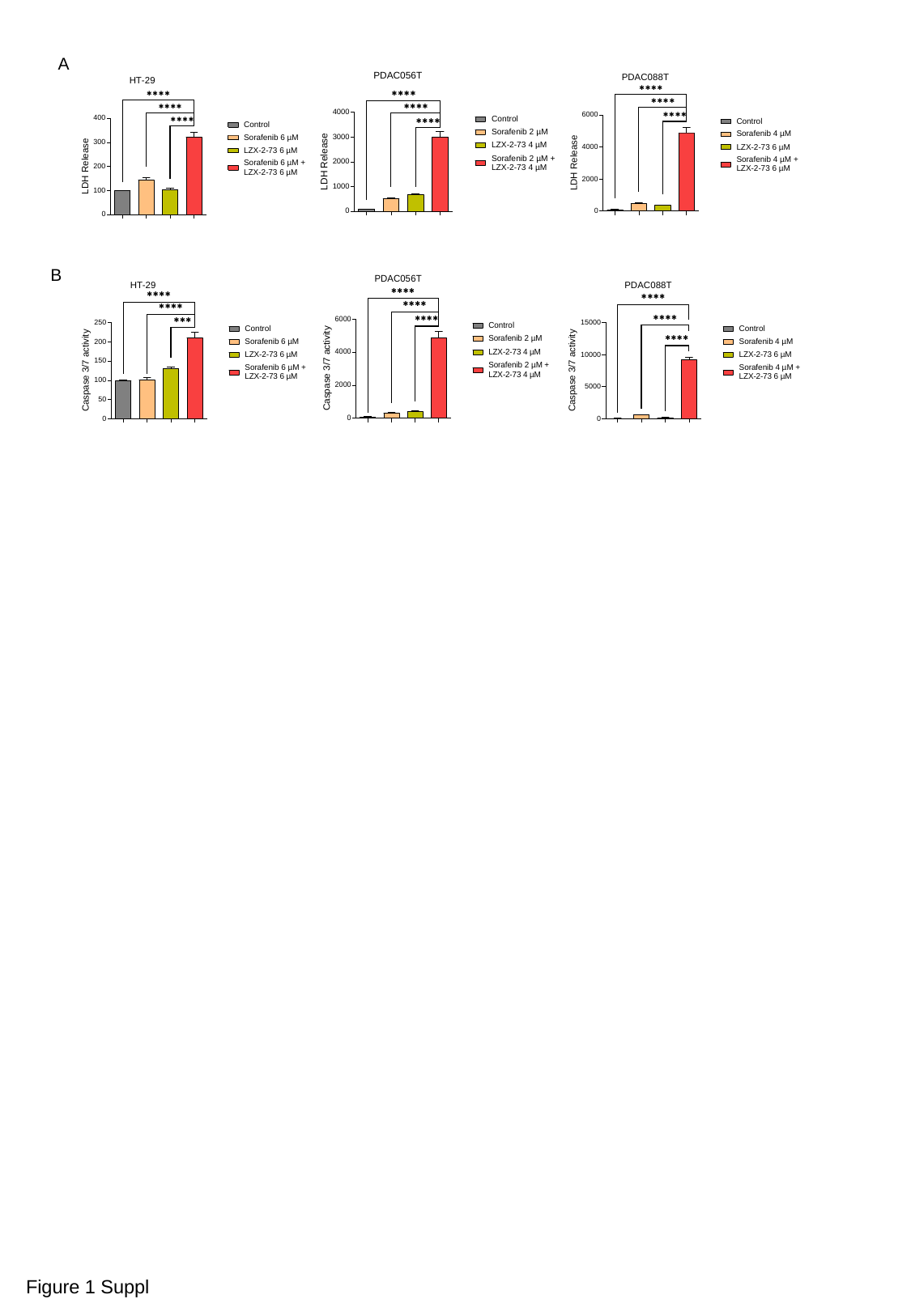

A
B
Figure 1 Suppl

Supplement: Supplementary file 1 — Supp Figure 1 [file 41419_2025_8178_MOESM1_ESM.pptx]

## Slide 1
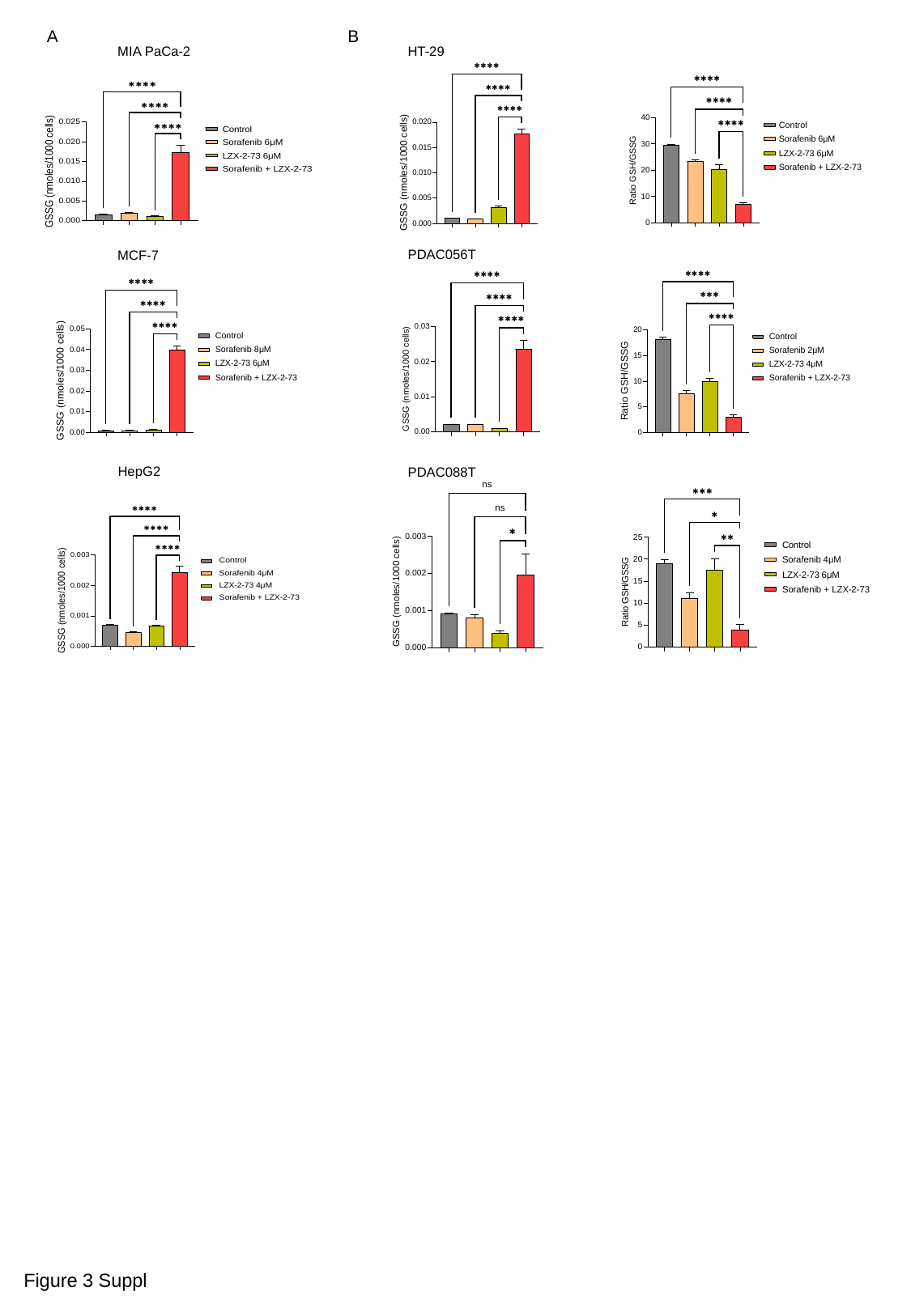

A
B
MIA PaCa-2
HT-29
PDAC056T
MCF-7
HepG2
PDAC088T
Figure 3 Suppl

Supplement: Supplementary file 3 — Supp Figure 3 [file 41419_2025_8178_MOESM3_ESM.pptx]
