# Supplementary material for: Synergistic antitumor activity of sorafenib and the NUPR1 inhibitor LZX-2-73 in multiple cancer models
Source: Cell Death Dis. 2025 Nov 17;16(1):839. doi: 10.1038/s41419-025-08178-8 (PMC12623841; doi:10.1038/s41419-025-08178-8)
Supplement: Supplementary file 2 — Supp Figure 2 [file 41419_2025_8178_MOESM2_ESM.pptx]

## Slide 1
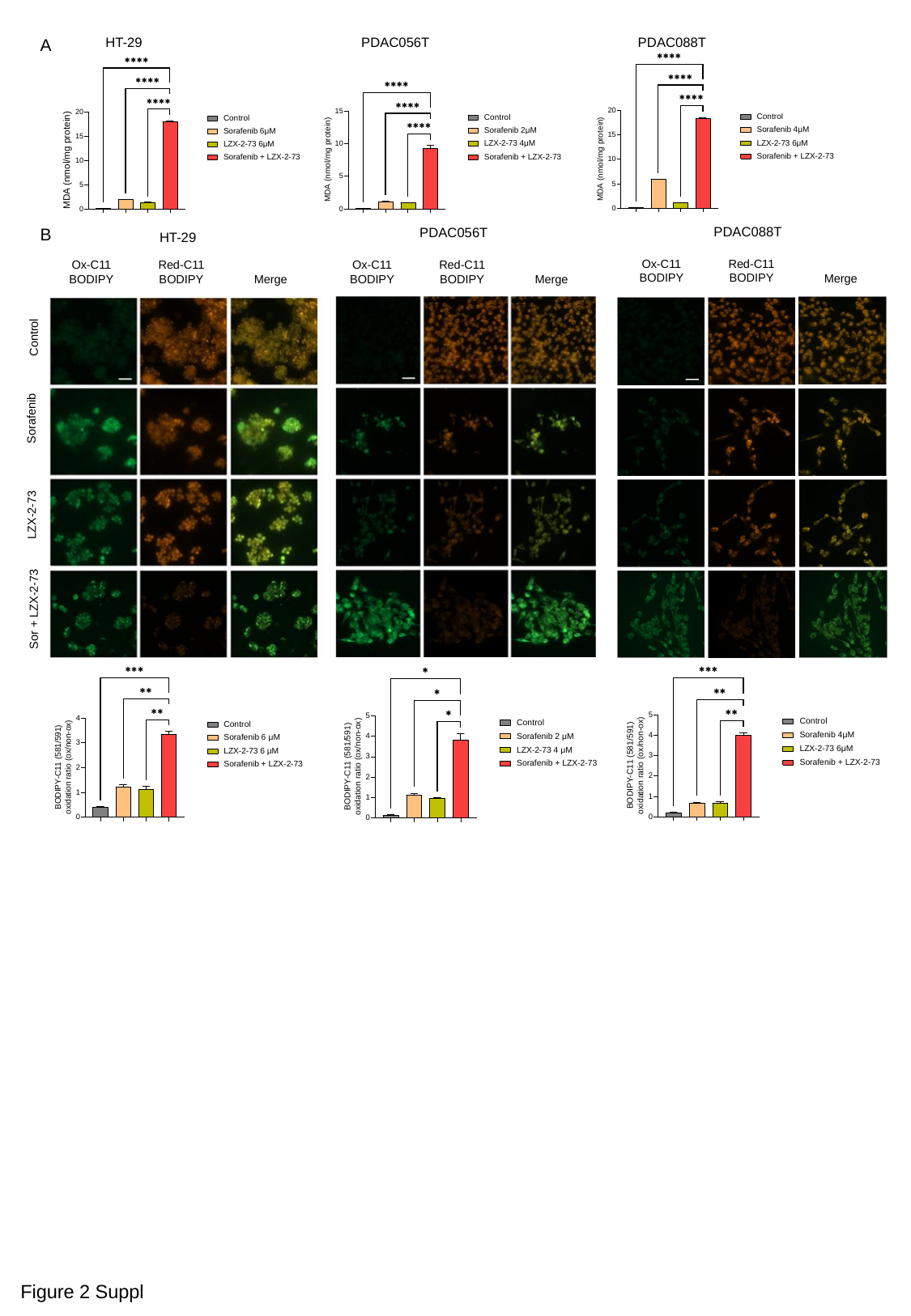

HT-29
PDAC056T
PDAC088T
A
B
PDAC088T
PDAC056T
HT-29
Ox-C11 BODIPY
Red-C11 BODIPY
Ox-C11 BODIPY
Red-C11 BODIPY
Ox-C11 BODIPY
Red-C11 BODIPY
Merge
Merge
Merge
Control
Sorafenib
LZX-2-73
Sor + LZX-2-73
Figure 2 Suppl
