# Supplementary material for: Synergistic antitumor activity of sorafenib and the NUPR1 inhibitor LZX-2-73 in multiple cancer models
Source: Cell Death Dis. 2025 Nov 17;16(1):839. doi: 10.1038/s41419-025-08178-8 (PMC12623841; doi:10.1038/s41419-025-08178-8)
Supplement: Supplementary file 4 — Supp Figure 4 [file 41419_2025_8178_MOESM4_ESM.pptx]

## Slide 1
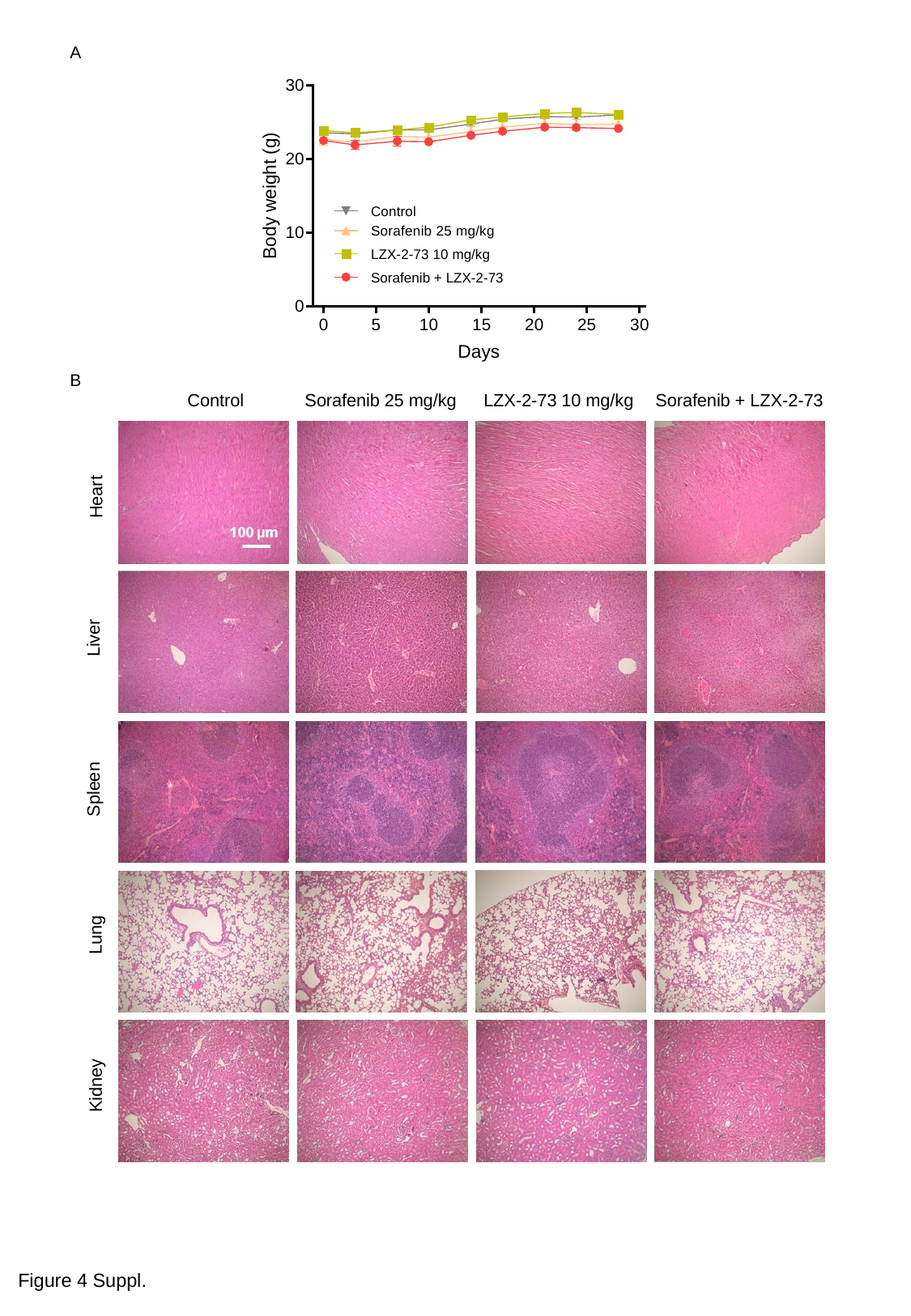

A
B
Control
Sorafenib 25 mg/kg
LZX-2-73 10 mg/kg
Sorafenib + LZX-2-73
Heart
Liver
Spleen
Lung
Kidney
Figure 4 Suppl.
