# Supplementary material for: Synergistic antitumor activity of sorafenib and the NUPR1 inhibitor LZX-2-73 in multiple cancer models
Source: Cell Death Dis. 2025 Nov 17;16(1):839. doi: 10.1038/s41419-025-08178-8 (PMC12623841; doi:10.1038/s41419-025-08178-8)
Supplement: Supplementary file 5 — Supp Figure 5 [file 41419_2025_8178_MOESM5_ESM.pptx]

## Slide 1
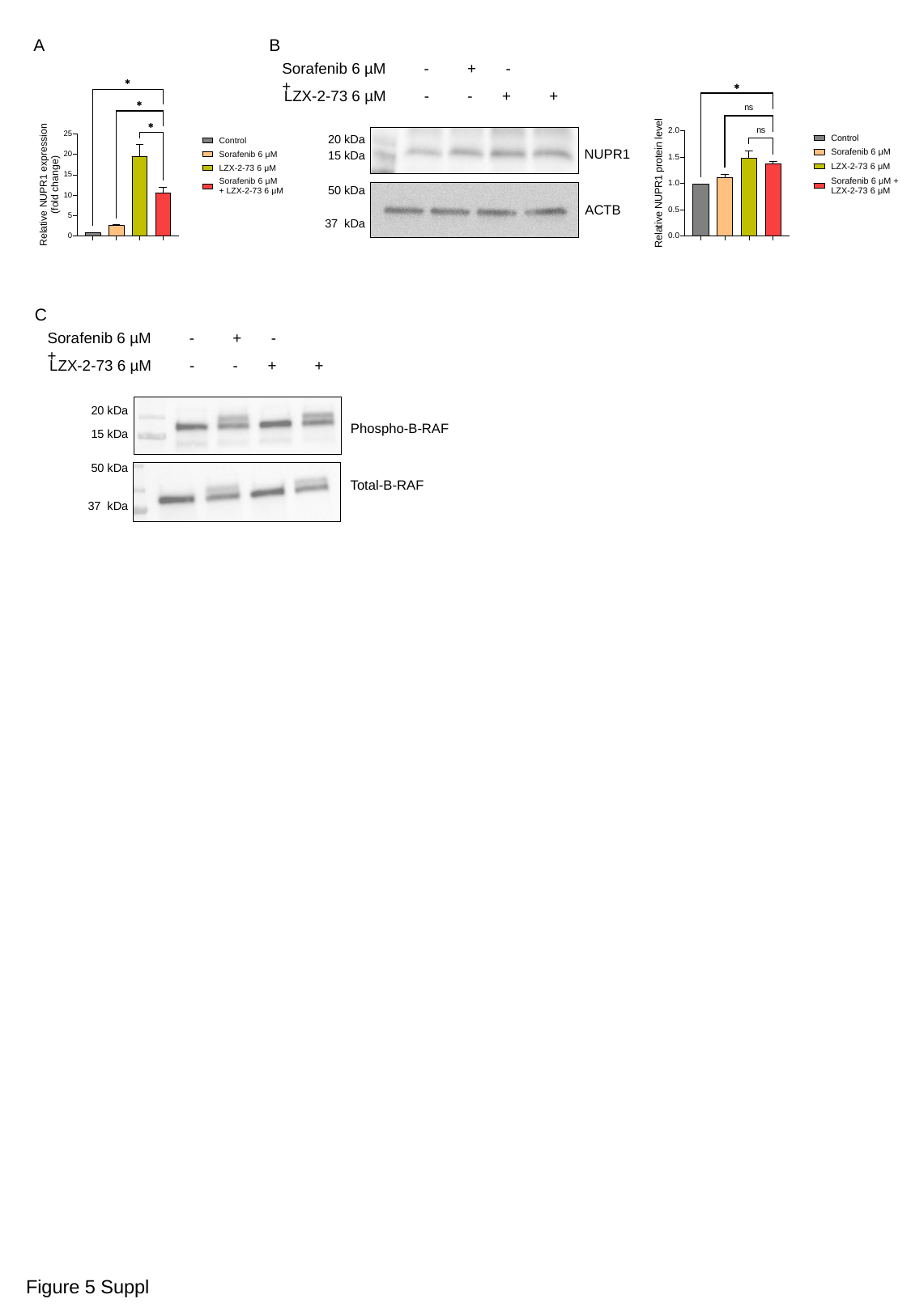

B
A
Sorafenib 6 µM - + - +
LZX-2-73 6 µM - - + +
20 kDa
NUPR1
15 kDa
50 kDa
ACTB
37 kDa
C
Sorafenib 6 µM - + - +
LZX-2-73 6 µM - - + +
20 kDa
Phospho-B-RAF
15 kDa
50 kDa
Total-B-RAF
37 kDa
Figure 5 Suppl
