# Supplementary material for: Synergistic antitumor activity of sorafenib and the NUPR1 inhibitor LZX-2-73 in multiple cancer models
Source: Cell Death Dis. 2025 Nov 17;16(1):839. doi: 10.1038/s41419-025-08178-8 (PMC12623841; doi:10.1038/s41419-025-08178-8)
Supplement: Supplementary file 6 — Legend of Supplementary Figures [file 41419_2025_8178_MOESM6_ESM.docx]

**Legend of Supplementary Figures**

**Supplementary Figure 1.**

**Combination of sorafenib and LZX-2-73 resulted in increased cell death in several tumor cell lines.** (A) LDH release and (B) caspase 3/7 activity measured on HT-29 cells, PDAC056T cells and PDAC088T cells. The cells were treated with single drugs and the drug combination for 48 hours. Data are shown as mean ± SEM. Statistical significance is indicated as follows: **** p < 0.0001 (1-way ANOVA, Dunnett’s test, compared to combinatory treatment) (n = 3).

**Supplementary Figure 2.**

**Combination of sorafenib and LZX-2-73 led to an increase of ROS accumulation in several tumor cell lines.** Levels of lipid peroxidation, indicated by MDA levels (A) or by the oxidation of the (B) BODIPY-C11 probe (measured via fluorescence microscopy to monitor the oxidized and non-oxidized variants of lipid peroxides), were assessed in cells treated with sorafenib, LZX-2-73, or the combination of both drugs for 72 hours on HT-29 cells, PDAC056T cells and PDAC088T cells. Statistical significance is indicated as follows: * p < 0.05, ** p < 0.01, **** p < 0.0001 (1-way ANOVA, Dunnett’s test, compared to combinatory treatment) (n = 3). Scale bar: 50 μm.

**Supplementary Figure 3.**

**Combination of sorafenib and LZX-2-73 depleted the antioxidative stress response in tumor cells.** (A) The GSSG content was measured in MIAPaCa-2 cells, MCF-7 cells and HepG2 cells treated with sorafenib, LZX-2-73 or the combination of the two drugs for 72 h. (B) The ratio of GSH/GSSG and total GSSG were measured in HT-29 cells, PDAC056T cells and PDAC088T cells treated with sorafenib, LZX-2-73 or the combination of the two drugs for 72 h. Statistical significance is indicated as follows: * p < 0.05, ** p < 0.01, **** p < 0.0001 (1-way ANOVA, Dunnett’s test, compared to combinatory treatment) (n = 3).

**Supplementary Figure 4.**

**Combined sorafenib and LZX-2-73 treatment in a tumor xenograft mouse model induced no significant toxicity.** Female Crl:NU(Ico)-*Foxn1^nu^* mice implanted with pancreatic cancer MIAPaCa-2 cell lines xenografts and treated daily with sorafenib (25 mg/kg), LZX-2-73 (10 mg/kg), or a combination of both drugs (25 mg/kg + 10 mg/kg) via intraperitoneal injection. (A) Body weight volume were measured twice per week. (B) Representative images of tissue sections following hematoxylin and eosin (H&E) staining of major organs from mice treated with sorafenib (25 mg/kg), LZX-2-73 (10 mg/kg), or their combination. Scale bar: 100 μm.

**Supplementary Figure 5.**

**Effect of LZX-2-73 and Sorafenib on NUPR1 expression in MIAPaCa-2 cells.** (A) qRT-PCR analysis of NUPR1 mRNA levels in MIAPaCa-2 cells treated for 24 hours with DMSO (control), sorafenib (6 µM), LZX-2-73 (10 µM), or a combination of both. Data are expressed as fold change relative to control, normalized to 36B4 (also known as RPLP0). Bars represent mean ± SEM of three independent experiments. P values were calculated using one-way ANOVA followed by Tukey’s post hoc test. (B) Representative Western blot showing NUPR1 protein levels under the same treatment conditions. b-actin was used as a loading control. The lower panel shows densitometric quantification of NUPR1 protein levels normalized to GAPDH, expressed as relative intensity compared to control. Bars represent mean ± SEM from at least three independent experiments. b-actin was used as a loading control. (C) Representative Western blot showing phospho B-RAF protein levels under the same treatment conditions. Total B-RAF was used as a loading control.
