# Supplementary material for: Synergistic antitumor activity of sorafenib and the NUPR1 inhibitor LZX-2-73 in multiple cancer models
Source: Cell Death Dis. 2025 Nov 17;16(1):839. doi: 10.1038/s41419-025-08178-8 (PMC12623841; doi:10.1038/s41419-025-08178-8)

Phospho-Nrf2 (The red box indicates the membrane used in the main figure. The first picture corresponds to the chemiluminescence signal, and the second one is the merge with the visible channel to show the molecular weight marker.)





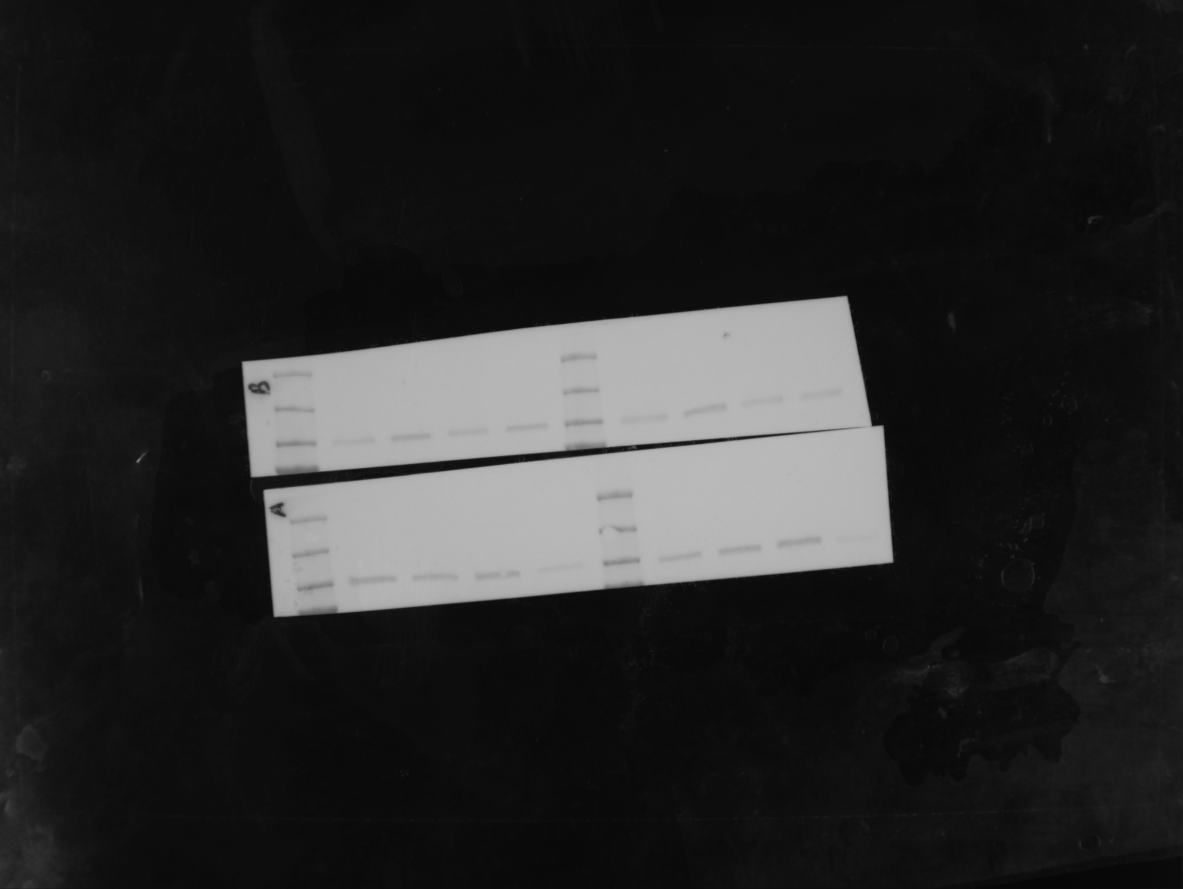


Total-NRF2 (The red box indicates the membrane used in the main figure. The first picture corresponds to the chemiluminescence signal, and the second one is the merge with the visible channel to show the molecular weight marker.)


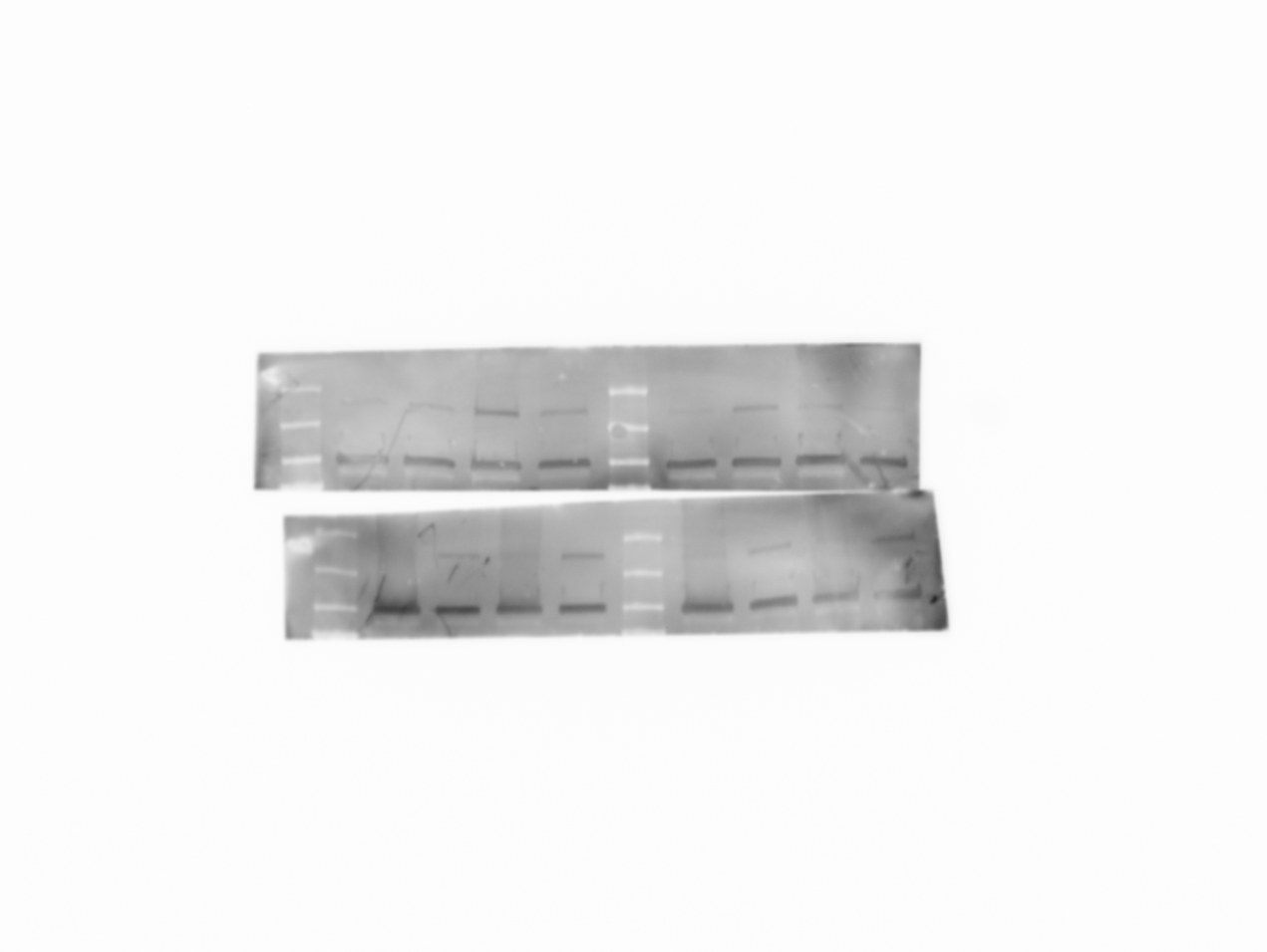


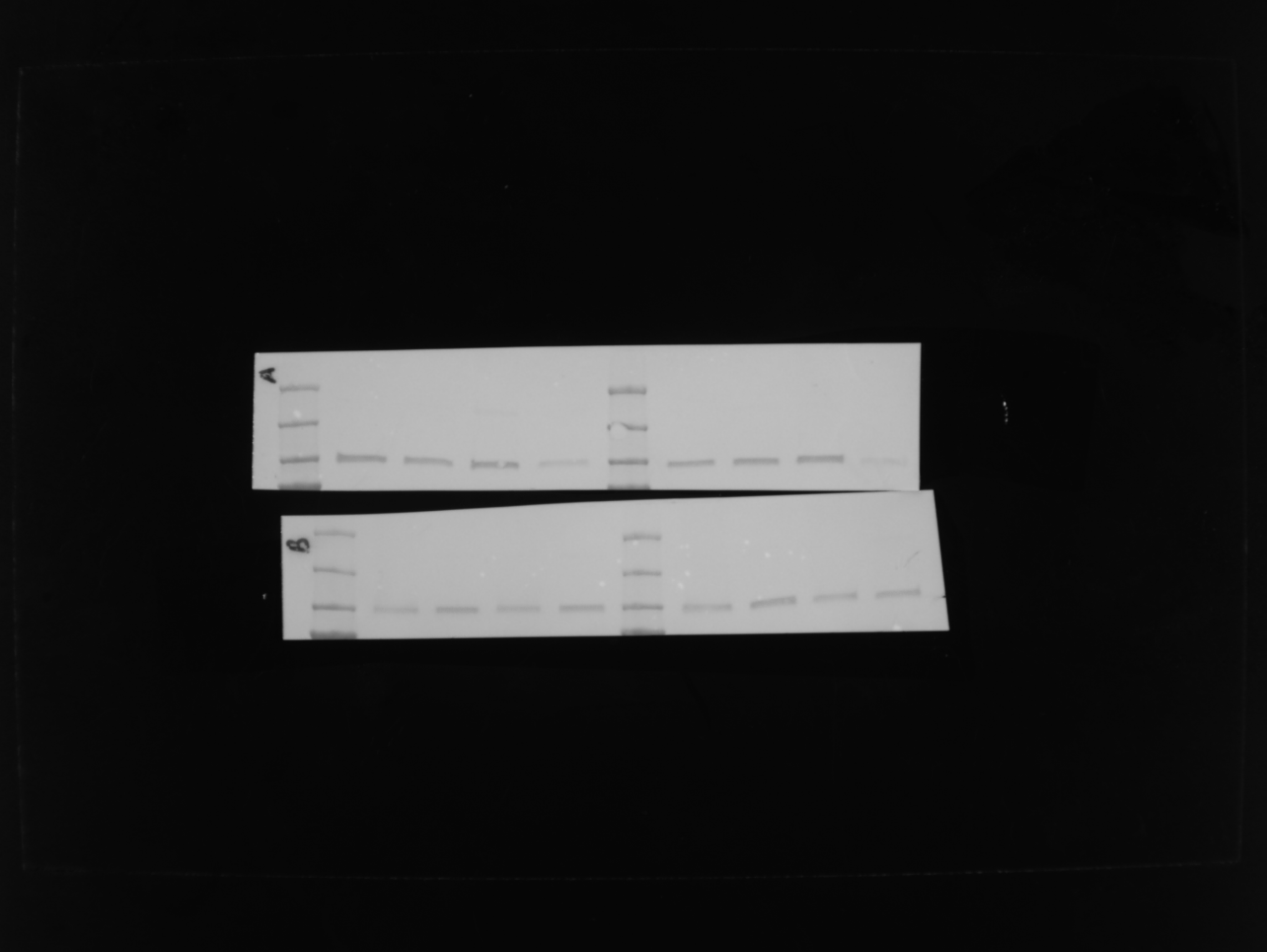


XCT (The red box indicates the membrane used in the main figure. The first picture corresponds to the chemiluminescence signal, and the second one is the merge with the visible channel to show the molecular weight marker.)


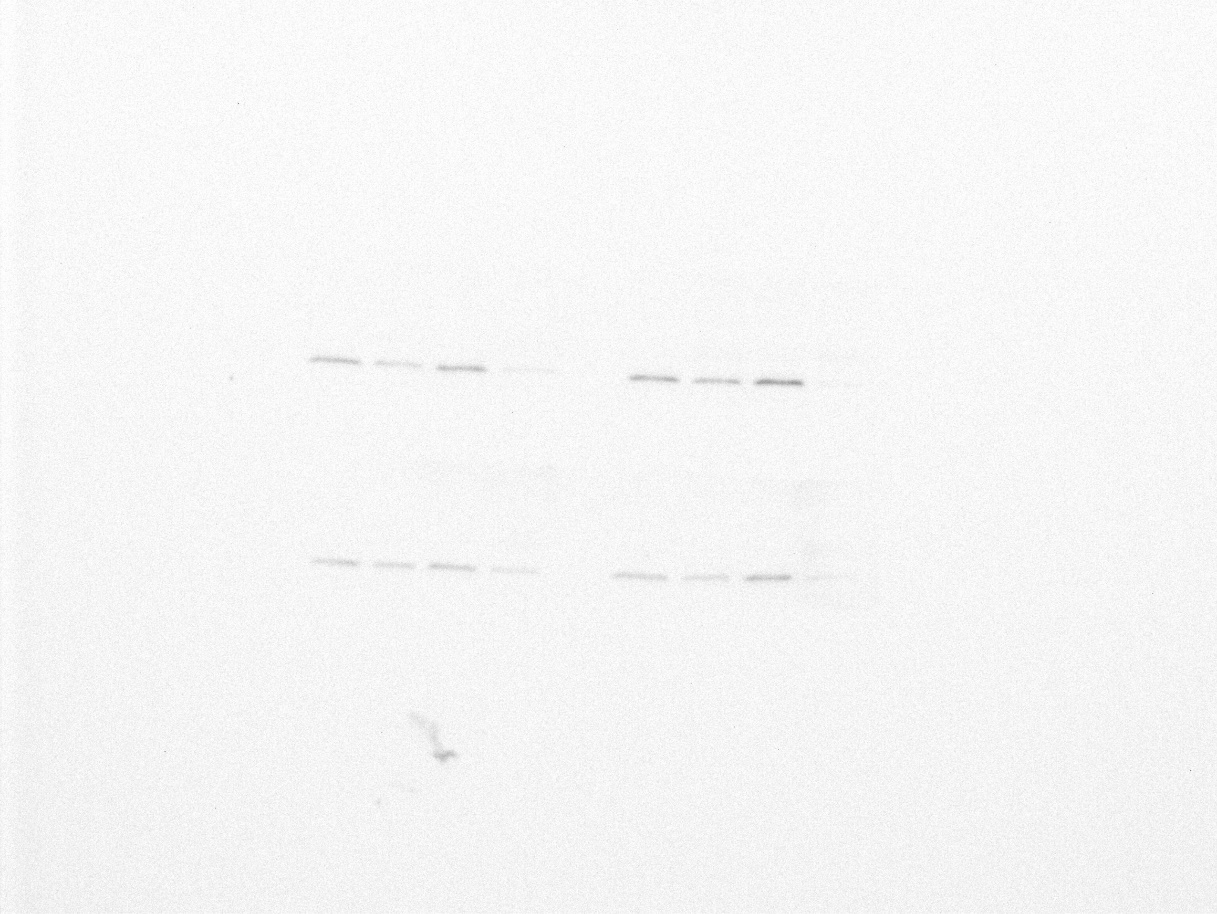

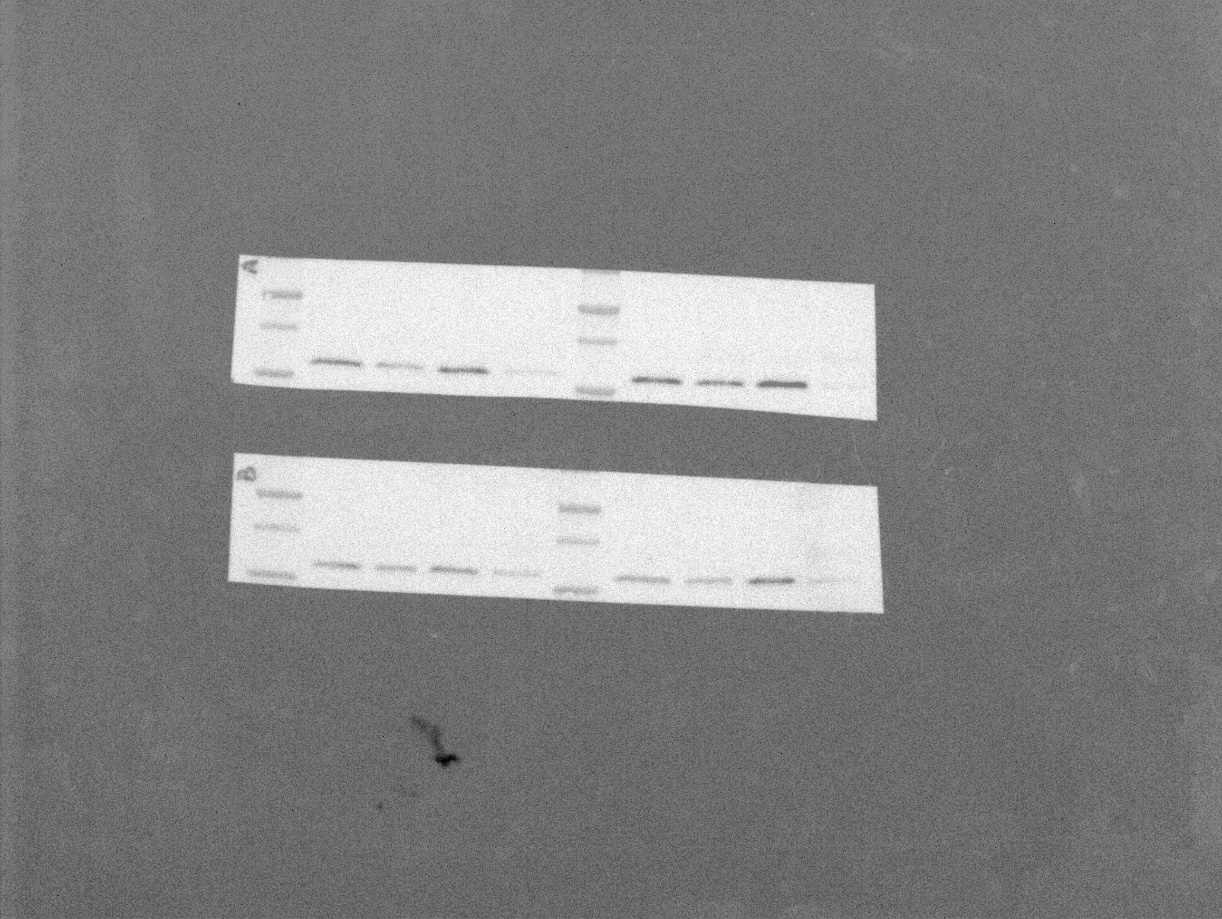


GPX-4 (The red box indicates the membrane used in the main figure. The first picture corresponds to the chemiluminescence signal, and the second one is the merge with the visible channel to show the molecular weight marker.)


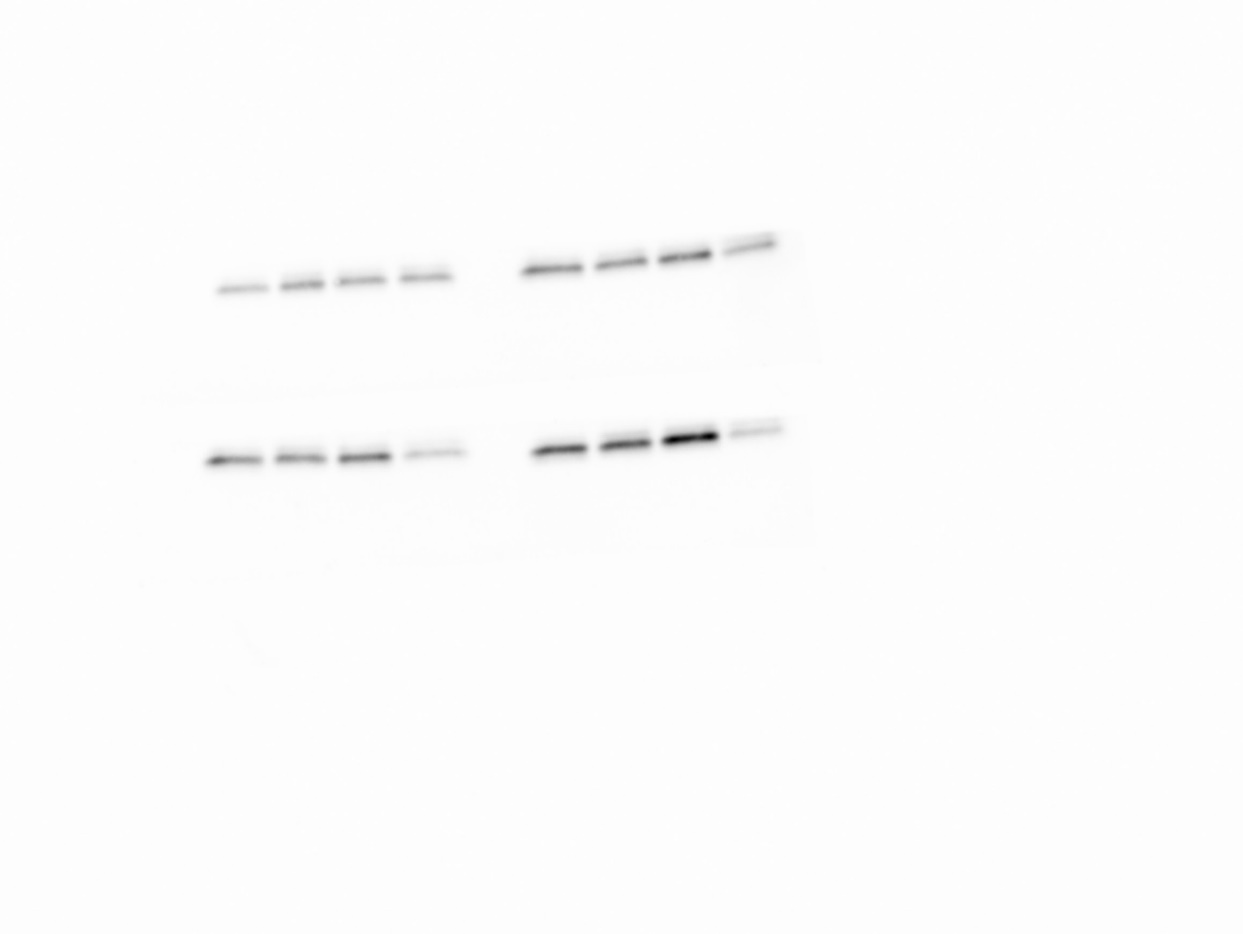




ACTB (The first picture corresponds to the chemiluminescence signal, and the second one is the merge with the visible channel to show the molecular weight marker.)


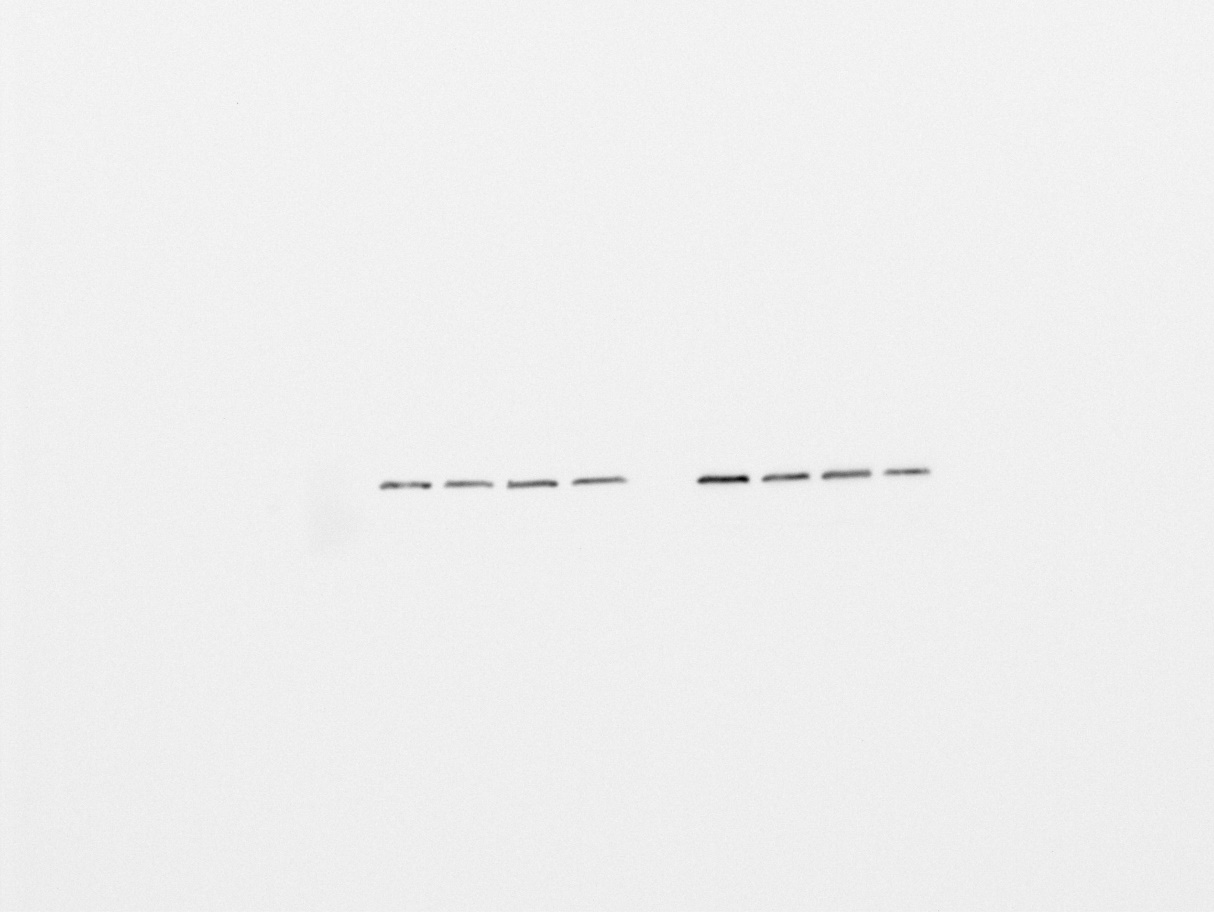




SURVIVIN


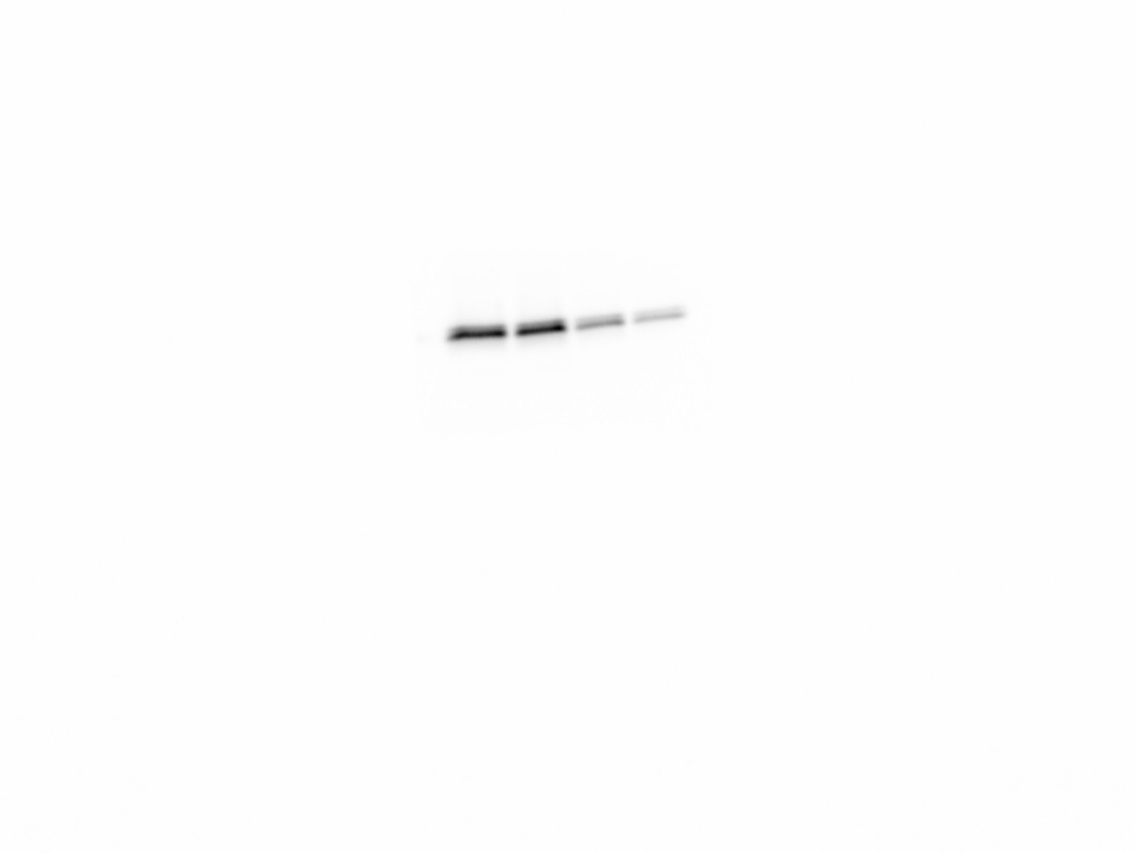




ACTB


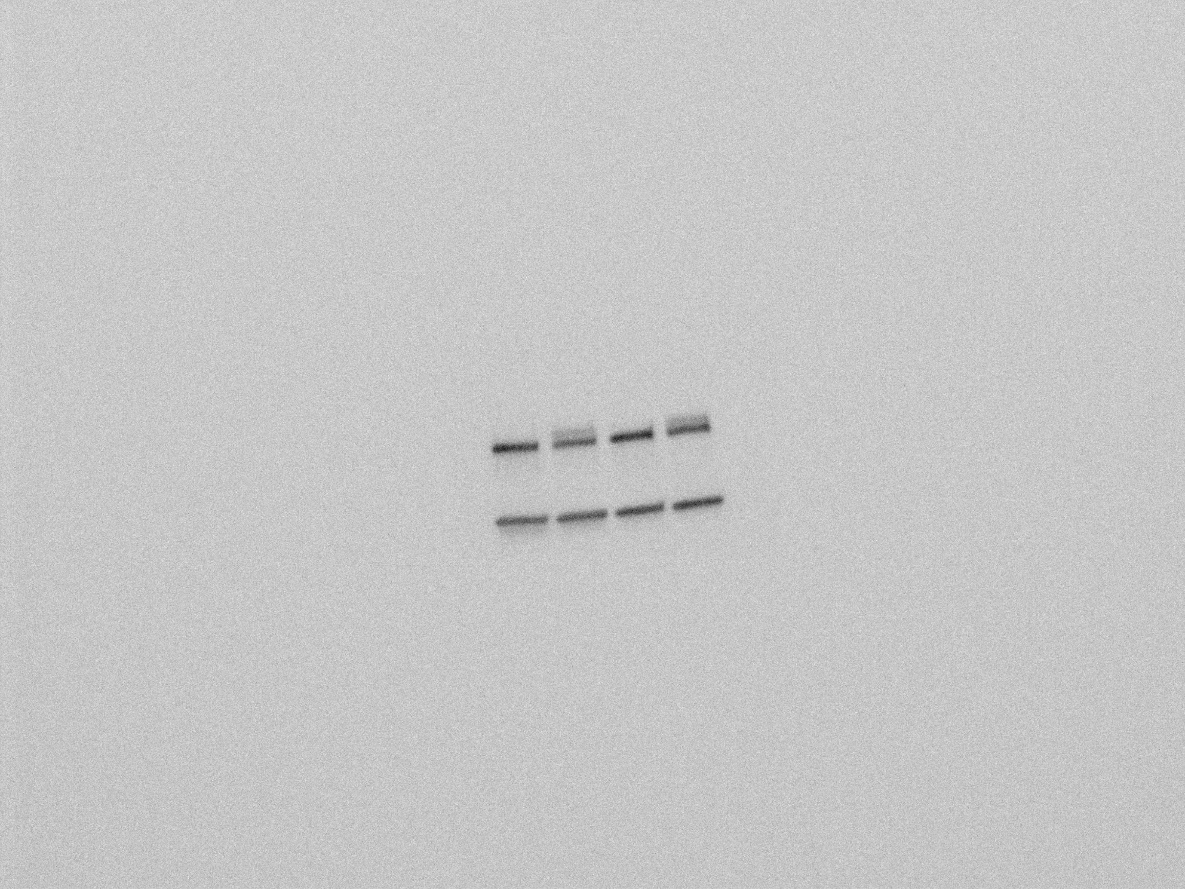

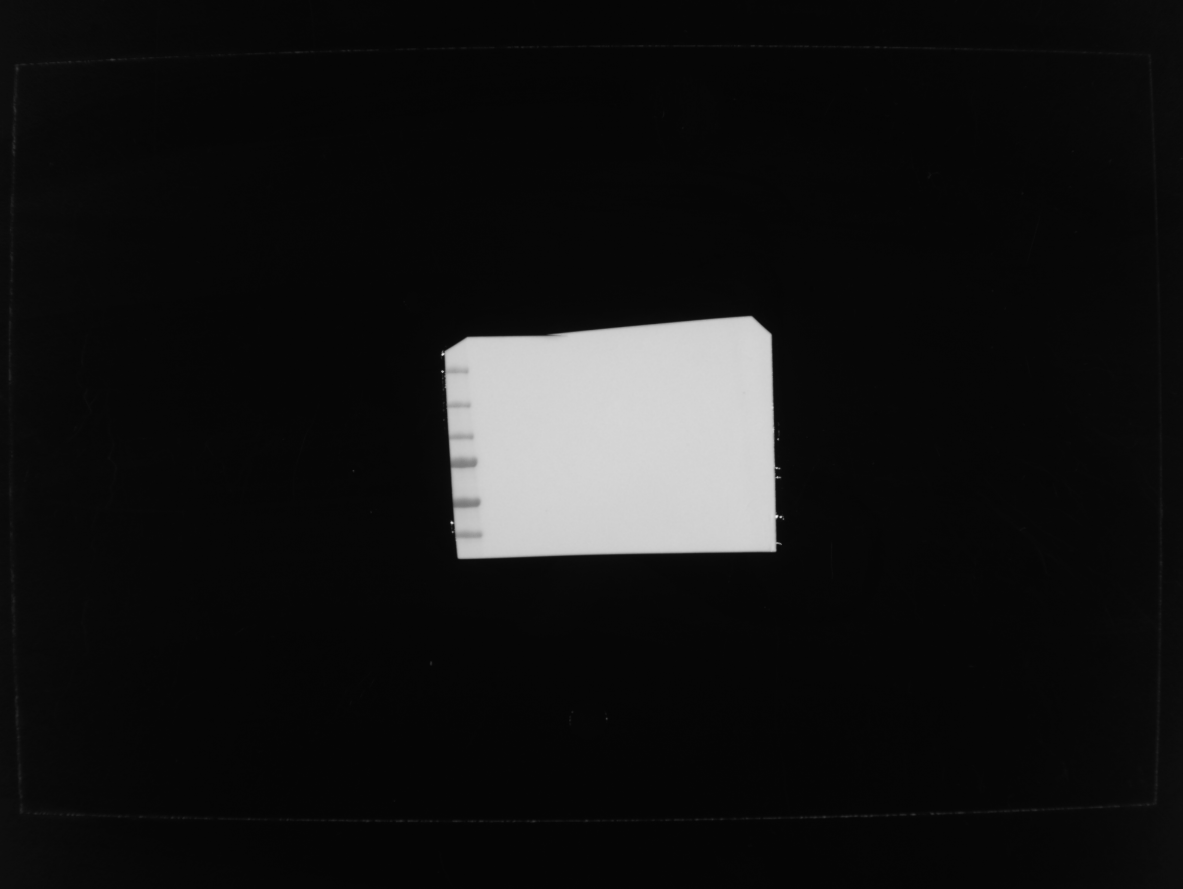


NUPR1


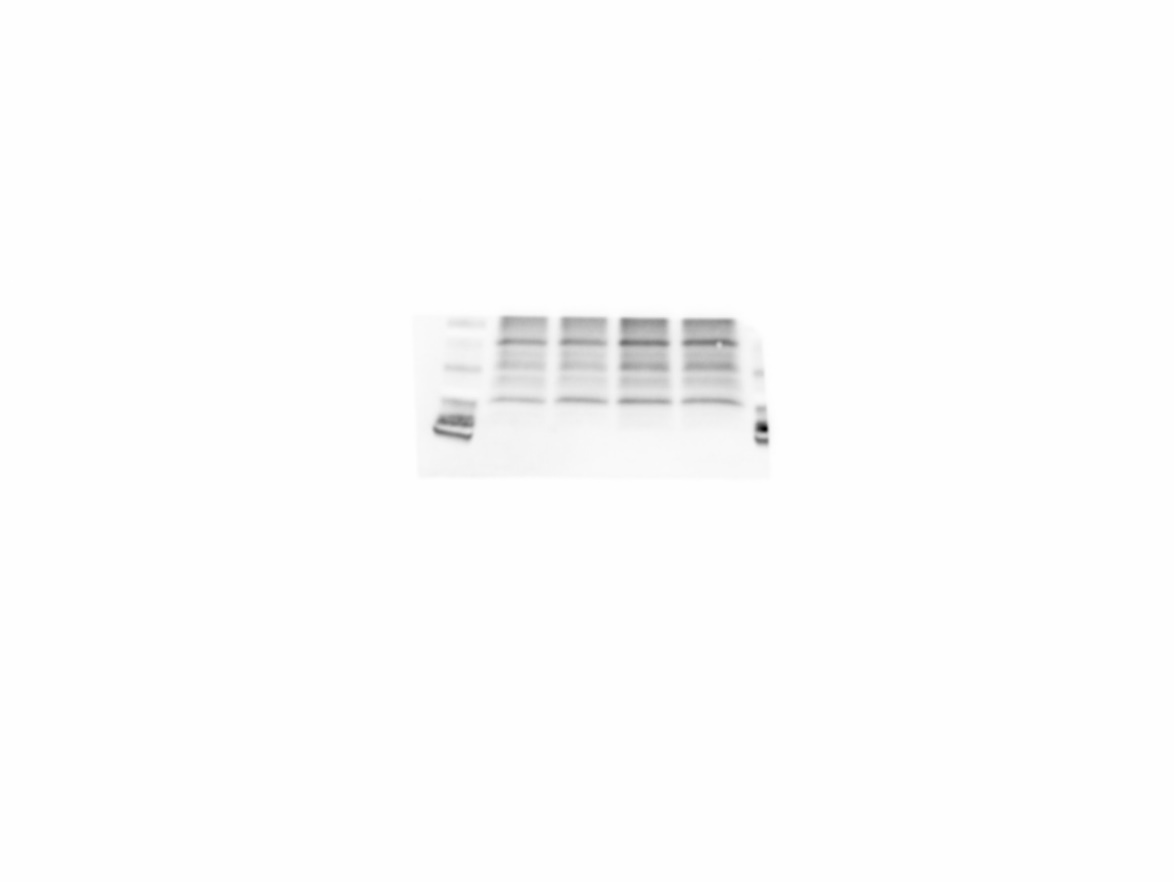




ACTB


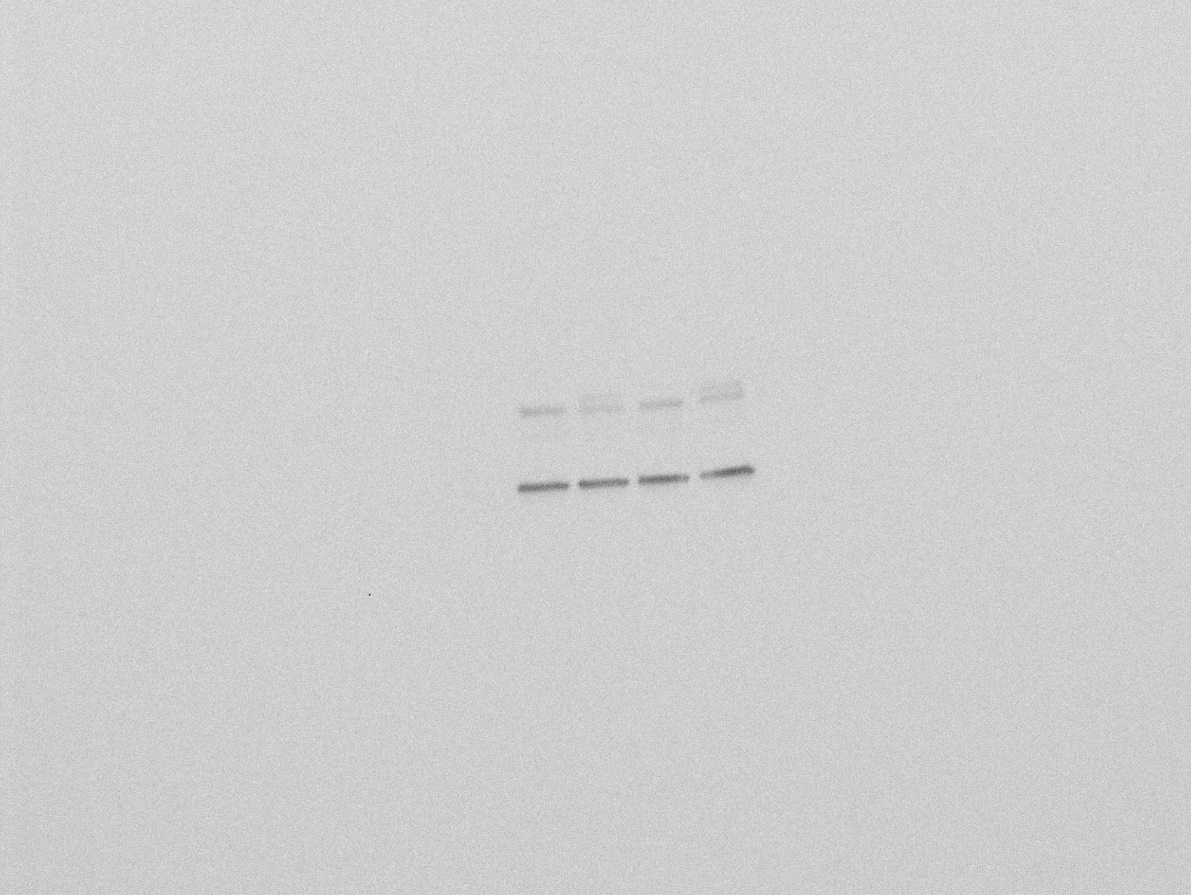

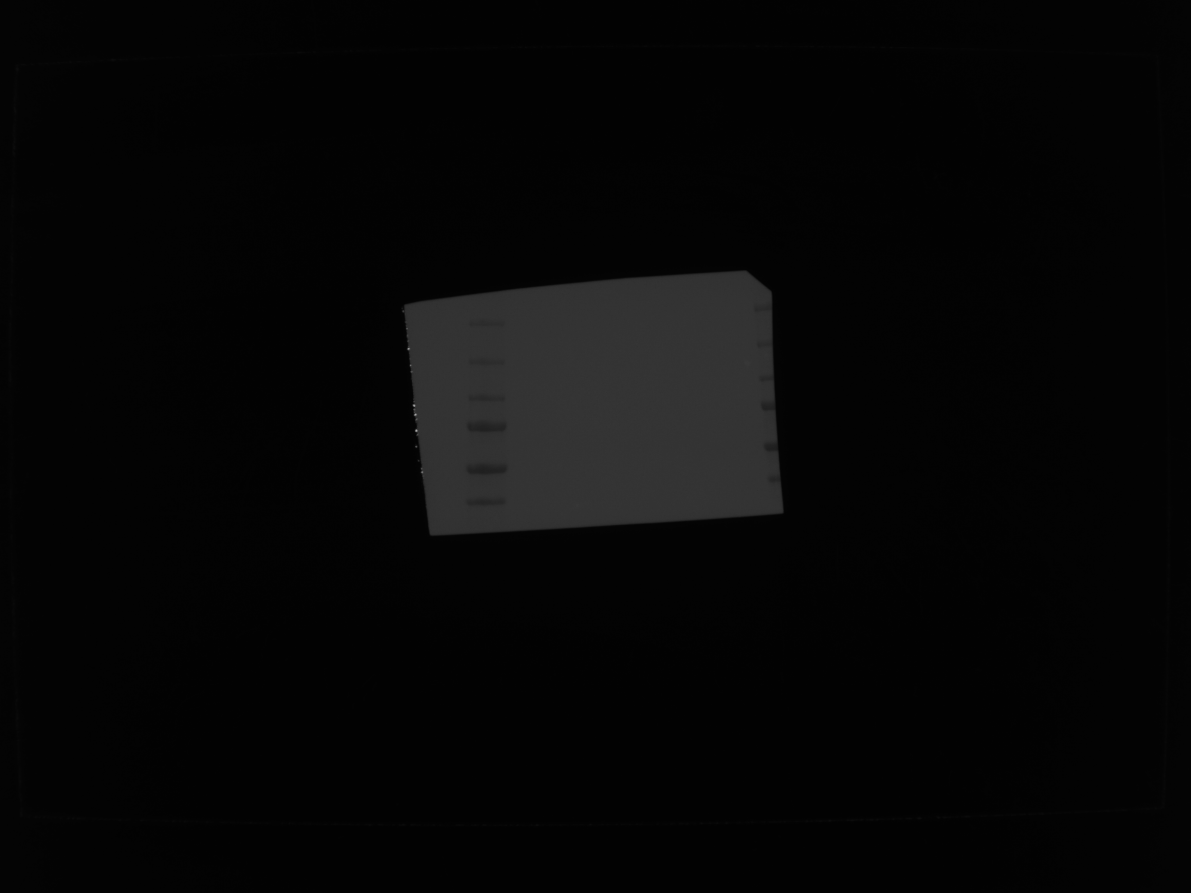


B-Raf


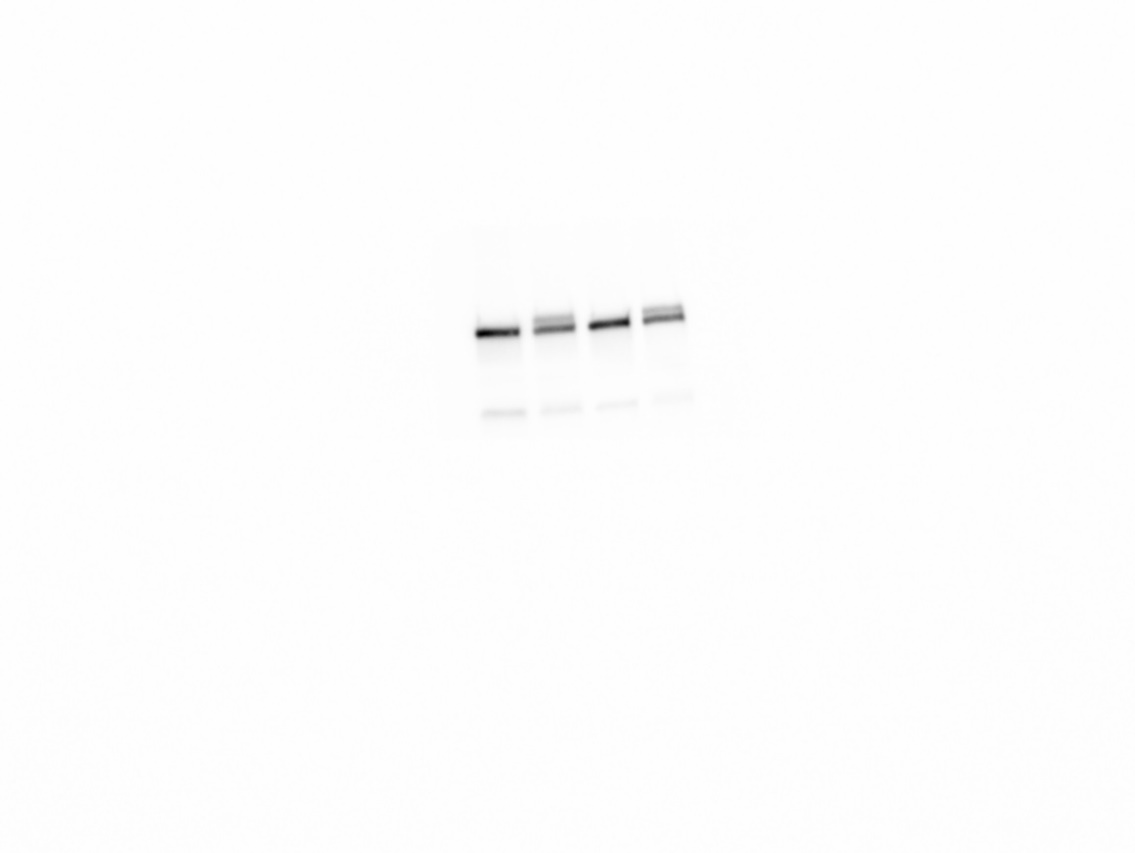




Phospho-B-Raf
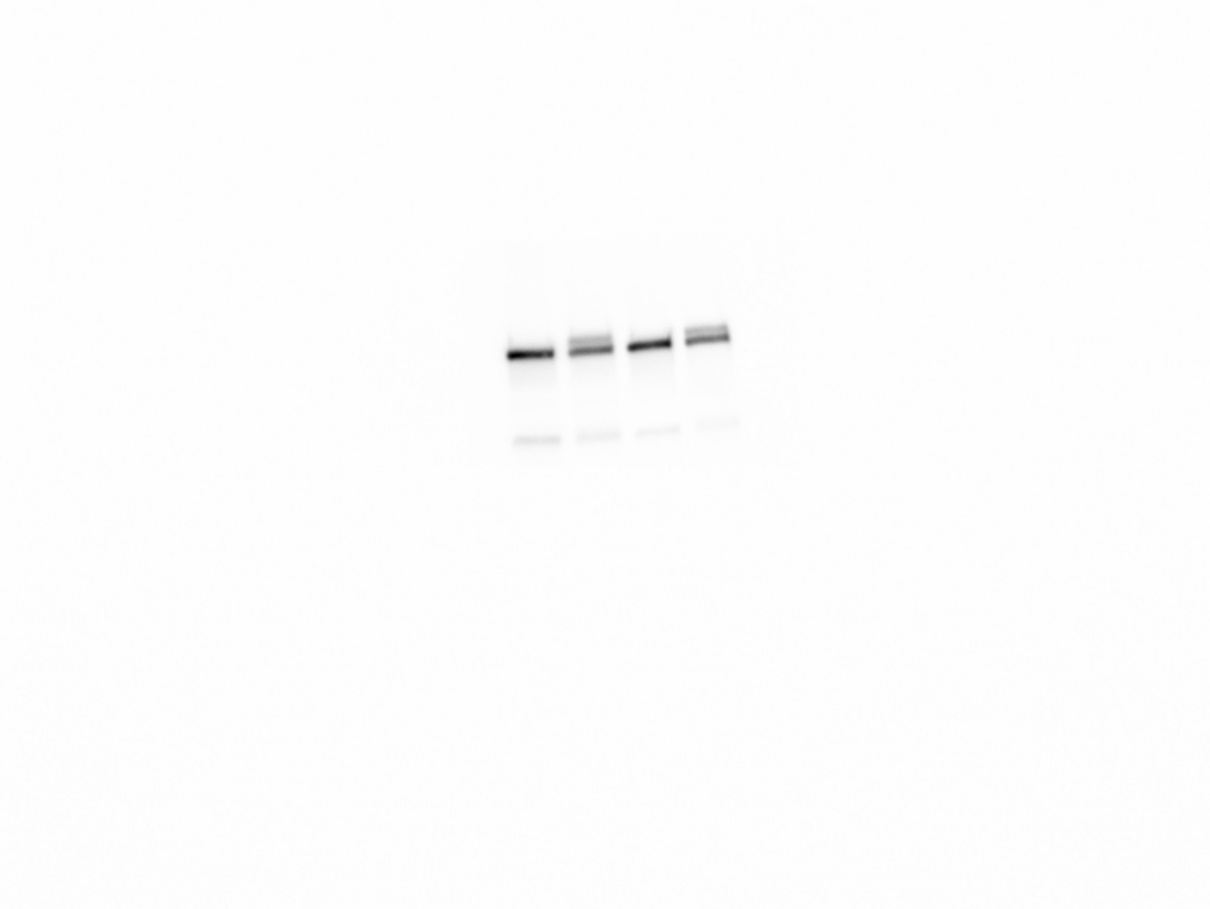

Supplement: Supplementary file 7 — Original blots uncropped [file 41419_2025_8178_MOESM7_ESM.docx]
